# Synthesis and In Vivo Antifungal Evaluation of 3-Acyl-bromoindole Regioisomers: A Multi-Targeting Study on Postharvest Pathogen Control and Molecular Dynamics

**DOI:** 10.3390/antibiotics15080801

**Published:** 2026-08-18

**Authors:** Alejandro Madrid, Valentina Silva, Katy Díaz, Evelyn Muñoz, David Cabezas, Karel Mena-Ulecia, Iván Montenegro, Carmina Sirignano, Enrique Werner, Ximena Besoain

**Affiliations:** 1 Laboratorio de Productos Naturales y Síntesis Orgánica (LPNSO), Facultad de Ciencias Naturales y Exactas, Universidad de Playa Ancha, Leopoldo Carvallo 270, Playa Ancha, Valparaiso 2340000, Chile; silvapedrerosv@gmail.com; 2 Laboratorio de Pruebas Biológicas, Departamento de Química, Universidad Técnica Federico Santa María, Av. España N1680, Valparaiso 2340000, Chile; katy.diaz@usm.cl; 3 Departamento de Química Orgánica, Facultad de Ciencias Químicas, Universidad de Concepción, Concepcion 4070371, Chile; emunoznu@udec.cl; 4 Centro de Investigación Nucleares para Aplicaciones en Salud y Biomedicina, División de Investigación y Aplicaciones Nucleares (CINASB), Comisión Chilena de Energía Nuclear, Nueva Bilbao 12051, Las Condes, Santiago 7550000, Chile; david.cabezas@cchen.cl; 5 Departamento de Ciencias Biológicas y Químicas, Facultad de Recursos Naturales, Universidad Católica de Temuco, Temuco 4810399, Chile; kmena@uct.cl; 6 Núcleo de Investigación en Bioproductos y Materiales Avanzados (BioMa), Vicerectoría de Investigación y Postgrado, Universidad Católica de Temuco, Temuco 4810399, Chile; 7 Center of Interdisciplinary Biomedical and Engineering Research for Health (MEDING), Escuela de Obstetricia y Puericultura, Facultad de Medicina, Universidad de Valparaíso, Angamos 655, Reñaca, Vina del Mar 2520000, Chile; ivan.montenegro@uv.cl; 8 Department of Pharmacy, School of Medicine and Surgery, University of Naples Federico II, Via D. Montesano 49, 80131 Naples, Italy; carmina.sirignano@unina.it; 9 Departamento de Ciencias Básicas, Campus Fernando May, Universidad del Bío-Bío, Avda. Andrés Bello 720, Casilla 447, Chillan 3780000, Chile; ewerner@ubiobio.cl; 10 Escuela de Agronomía, Facultad de Ciencias Agronómicas y de los Alimentos, Pontificia Universidad Católicade Valparaíso, San Francisco s/n La Palma, Quillota 2260000, Chile; ximena.besoain@pucv.cl

**Keywords:** microwave-assisted, indole derivatives, *Botrytis cinerea*, *Monilinia fructicola*

## Abstract

**Background/Objectives**: Postharvest fungal decay caused by *Botrytis cinerea* and *Monilinia fructicola* poses major threats to global fruit security. Driven by the need for sustainable crop protection agents, this work presents the systematic synthesis, biological evaluation, and computational modeling of a comprehensive 33-compound library of 3-acyl-bromoindole regioisomers (series **4a–k**, **5a–k**, and **6a–k**) to establish clear structure–activity relationship (SAR) design rules. **Methods**: The regioisomeric library was assembled via a microwave-assisted catalytic protocol in an ionic liquid, expanding the known chemical space with seven newly synthesized 4-bromoindole derivatives (**4d–f**, **4h–k**). Primary in vitro data were modeled using Hansch QSAR and Principal Component Analysis (PCA). Postharvest in vivo efficacy was evaluated on fresh ‘Lapins’ sweet cherries inoculated with *M. fructicola*. Molecular docking and 100 ns molecular dynamics (MD) simulations were performed against succinate dehydrogenase (SDH) and *M. fructicola* catalase 2 (MfCat2). **Results**: In vitro screening demonstrated marked target selectivity: parent core **4** displayed high mycelial suppression against *M. fructicola* (EC_50_ = 7.05 µg/mL), whereas C3-acylation with a four-carbon linear chain (**4c**) achieved optimal broad-spectrum dual action (98% and 86% spore germination inhibition). In vivo cherry bioassays proved that bromoindoles **4**, **6a**, and **6d** significantly suppressed Brown Rot severity to 44–47% (a 20–27% reduction vs. untreated control). Docking and MD trajectories confirmed stable multi-target binding within SDH and MfCat2 active sites (RMSD < 2.0 Å). **Conclusions**: Bromine regiochemistry dictates pathogen selectivity and life-stage targeting. The novel 4-bromoindole derivatives and multi-target profile establish these scaffolds as promising leads for postharvest crop protection.

## 1. Introduction

Postharvest fungal diseases caused by pathogens such as *Botrytis cinerea* and *Monilinia fructicola* represent one of the most significant threats to food security and the global agricultural economy [1]. *B. cinerea*, known for its broad host range, and *M. fructicola*, the primary cause of brown rot in stone fruits, drastically reduce crop yields and compromise fruit quality during storage and transport [2,3,4]. Although conventional synthetic fungicides remain the primary control strategy, the rapid emergence of resistant strains, especially toward single-site inhibitors such as succinate dehydrogenase inhibitors (SDHIs), and increasing environmental restrictions have driven an urgent search for new antifungal agents with more efficient and diverse mechanisms of action [5,6,7,8]. In this context, the indole nucleus has emerged as a privileged scaffold in agricultural chemistry due to its structural versatility and wide range of biological activities [9]. Recent investigations by our group have systematically explored how halogenation on the indole ring influences bioactivity [10,11]. Specifically, our group previously observed that 3-acyl-6-bromoindole derivatives display promising fungicidal activity, which in silico modeling suggested was tied to potential binding within succinate dehydrogenase (SDH) [10]. Subsequently, we demonstrated that the 3-acyl-5-bromoindole series yielded compounds with appreciable capacity to inhibit the conidial germination of *M. fructicola*, a mechanism proposed to involve interactions with catalase 2 (MfCat2) [11].

The strategic selection of the 3-acyl-indole scaffold is grounded in its ability to mimic naturally occurring bioactive molecules and its capacity to establish key hydrogen-bonding interactions within the active sites of fungal enzymes [11,12,13]. The introduction of an acyl group at the C3 position significantly enhances the lipophilicity and molecular rigidity of the indole core, facilitating its penetration through the complex fungal cell wall and increasing its affinity for the hydrophobic pockets of targets such as SDH [10,14,15]. Furthermore, the presence of a bromine atom on the benzenoid ring is critical; halogens are known to participate in unique halogen-bonding interactions and modulate the electronic density of the indole system via inductive effects [16,17]. Despite these advances, the systematic impact of the bromine atom’s position on biological activity has not been comprehensively explored across the full 4-, 5-, and 6-substitution map. Literature and our previous findings suggest that slight changes in the substitution pattern of the indole ring can drastically alter physicochemical properties, such as lipophilicity and molecular volume, dictating biological potency and selectivity toward molecular targets [18,19,20]. This positional dependence phenomenon has been widely observed in other bioactive scaffolds of agricultural relevance, such as chalcones, polyphenols, and other heterocycles, where electronic redistribution and steric hindrance resulting from halogen substitution drastically modulate the ability of these molecules to interact with specific active-site targets [21,22,23,24]. Therefore, a comparative study including the 4-position, previously unexplored in our series, is necessary to establish clear Structure–Activity Relationship (SAR) design rules for new multi-target fungicides.

In the present work, we address this knowledge gap by reporting an efficient microwave-assisted catalytic methodology for the synthesis of a comprehensive 33-compound library of 3-acyl-bromoindole regioisomers (4, 5, and 6-positions), expanding the known chemical space with seven newly synthesized 4-bromoindole derivatives (**4d**, **4e**, **4f**, **4h**, **4i**, **4j**, and **4k**). Compared to traditional thermal acylation protocols, which often require extended reaction times (several hours) and harsh reaction conditions, this microwave-assisted approach using yttrium triflate (Y(OTf)_3_) in the ionic liquid [BMI]BF_4_ provides a highly regioselective, expedited (5–40 min), and sustainable route to afford these target regioisomers in high purity [10,11]. To determine their biological potential, in vitro antifungal assays were conducted for the entire library against both *B. cinerea* and *M. fructicola*, evaluating their impact on mycelial growth and conidial germination. Furthermore, a SAR analysis was performed using Principal Component Analysis (PCA) and Hansch parameters to identify the key physicochemical descriptors governing their efficacy. To bridge the gap between laboratory results and agricultural application, we extended our investigation to a practical agricultural scenario by evaluating the in vivo antifungal efficacy of the most promising candidates on fresh ‘Lapins’ cherries inoculated with *M. fructicola*, quantifying decay through a standardized severity index. Finally, 100 ns molecular dynamics simulations were carried out to validate the stability of the multi-target binding of the most active candidates within the active sites of SDH and MfCat2, providing a molecular rationale for their observed antifungal potency against postharvest pathogens.

## 2. Results and Discussion

### 2.1. Microwave-Assisted Regioselective Synthesis of 3-Acyl-bromoindoles

**Scheme 1:**
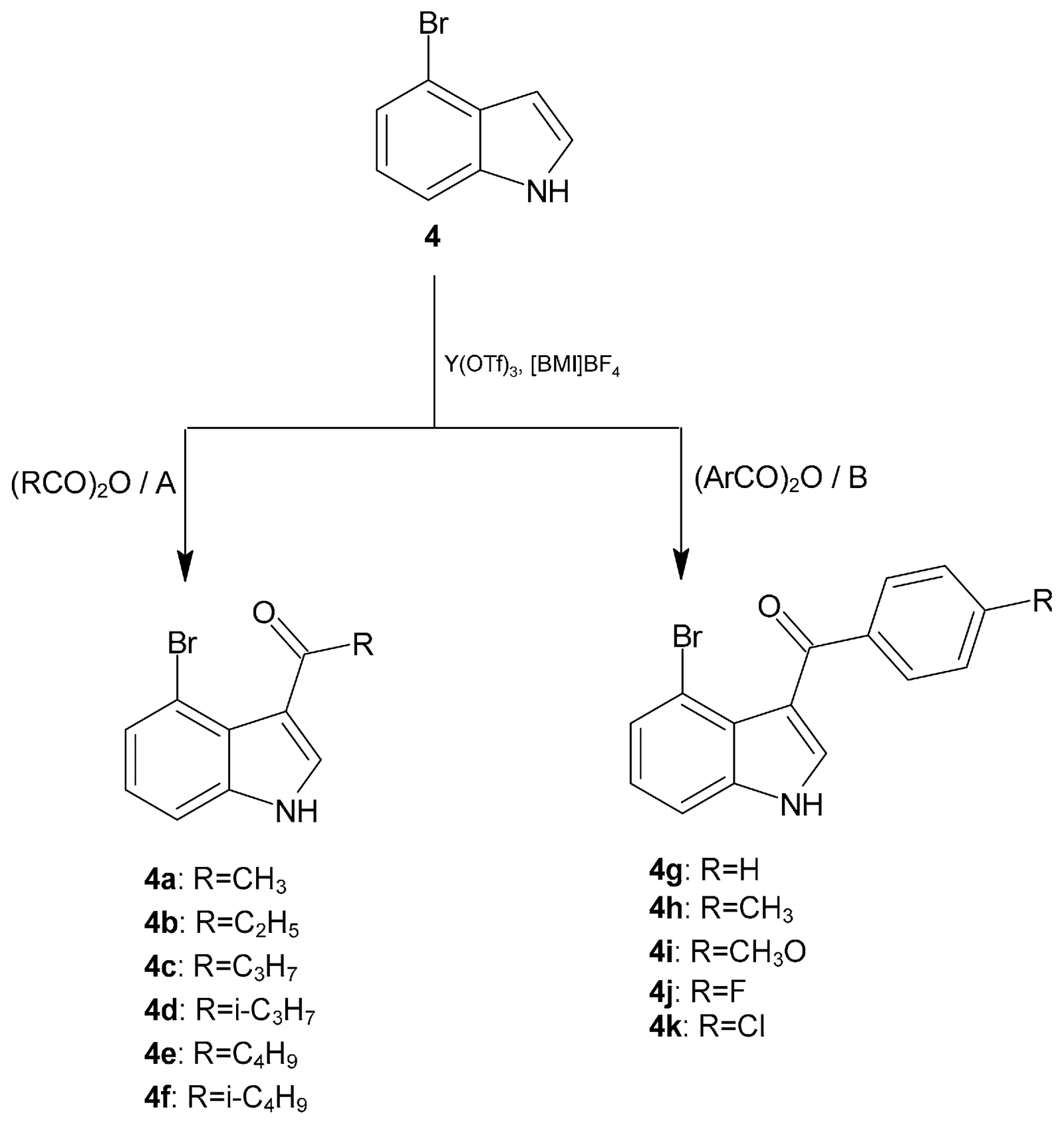
General synthetic model for the microwave-assisted C3-acylation of bromoindoles catalyzed by Y(OTf)_3_ in [BMI]BF_4_, illustrated for the 4-bromoindole series (**4a**–**k**). Condition A: Aliphatic anhydrides, MW irradiation (100 W), 70–90 °C, 5–15 min. Condition B: Aromatic anhydrides, MW irradiation (150 W), 90–120 °C, 20–40 min. Analogous synthetic pathways were applied for the 5- and 6-bromoindole regioisomeric series (**5a**–**k** and **6a**–**k**).

To evaluate the impact of bromine positioning on antifungal potency across the complete 4-, 5-, and 6-regioisomeric landscape, a unified catalytic protocol was implemented (Scheme 1). The C3-acylation of the bromoindole cores was achieved using yttrium triflate [(Y(OTf)_3_, 1 mol%] as the Lewis acid catalyst in the ionic liquid ([BMI]BF_4_) under microwave activation. The 4-bromoindole core (**4**) is depicted in Scheme 1 as a representative general model to illustrate the synthetic divergence dictated by the nature of the acylating agent. Unlike previously reported protocols for the 5- and 6-bromoindole series where a fixed microwave power was employed, the reaction parameters were further optimized in this work based on the electronic and steric profile of the anhydrides. Linear and branched aliphatic anhydrides proceeded smoothly under mild thermal settings and a reduced power output (Condition A: 100 W, 70–90 °C, 5–15 min), yielding the corresponding 3-acyl derivatives (**4a**–**f**, **5a**–**f**, and **6a**–**f**). Conversely, aromatic anhydrides required higher activation temperatures and energy input (Condition B: 150 W, 90–120 °C, 20–40 min) to overcome steric hindrance and resonance stabilization, efficiently affording benzoyl derivatives (**4g**–**k**, **5g**–**k**, and **6g**–**k**). When compared to conventional Friedel–Crafts acylation or traditional conductive heating methods, which typically require strong Lewis acids, volatile organic solvents, and prolonged reflux times ranging from several hours to overnight [25], this microwave-assisted protocol offers significant advantages. Using a catalytic amount of Y(OTf)_3_ (1 mol%) in the reusable ionic liquid under microwave irradiation dramatically accelerates the transformation (5–40 min) while providing complete C3-regioselectivity, preventing unwanted *N*-acylation or over-acylation, and yielding cleaner crude products with higher energy efficiency. Regarding synthetic efficiency, a clear regioisomeric dependence on isolated yields was observed, following the performance trend 5-bromoindole > 6-bromoindole > 4-bromoindole. The 5-bromoindole series consistently provided the highest conversion and isolated yields ([44.0–99.0]%), outperforming the 6-bromoindole derivatives ([28.8–87.5]%). In contrast, the 4-bromoindole series exhibited the lowest overall yields within the family ([20.9–80.6]%). Notably, within the 4-bromoindole series, seven derivatives are reported here for the first time (**4d**, **4e**, **4f**, **4h**, **4i**, **4j**, and **4k**), expanding the known chemical space of this regioisomeric family. This performance trend is driven by an interplay of electronic and steric factors. In the 5-bromo isomer, the halogen atom balances the polarization of the aromatic core without restricting access to C3 [26]. Conversely, substitution at the 6-position exerts a mild resonance-driven deactivation on C3 nucleophilicity, slightly slowing down the acylation rate compared to the 5-series [27,28]. Finally, the lower yields observed for the 4-bromo series are attributed to direct steric shielding imposed by the C4-bromine atom over the adjacent C3 reaction center [28,29,30]. Nevertheless, this unified microwave methodology served as a robust template to assemble the complete 33-compound regioisomeric library for subsequent biological and SAR analyses.

### 2.2. In Vitro Antifungal Activity

To evaluate the biological potential and the impact of bromine regiochemistry, the in vitro antifungal activity of the 4-bromoindole series (**4a–k**) and its parent compound (**4**) was evaluated against *B.* and *M. fructicola* (Table 1). These results were comparatively analyzed alongside previously reported data for the 5-bromoindole (**5a**–**k)** and 6-bromoindole (**6a**–**k**) series. The primary screening evaluated both vegetative mycelial growth inhibition (EC_50_) and conidial germination inhibition (ICG%), demonstrating clear differences in antifungal activity among the distinct regioisomeric families.

**Figure 1:**
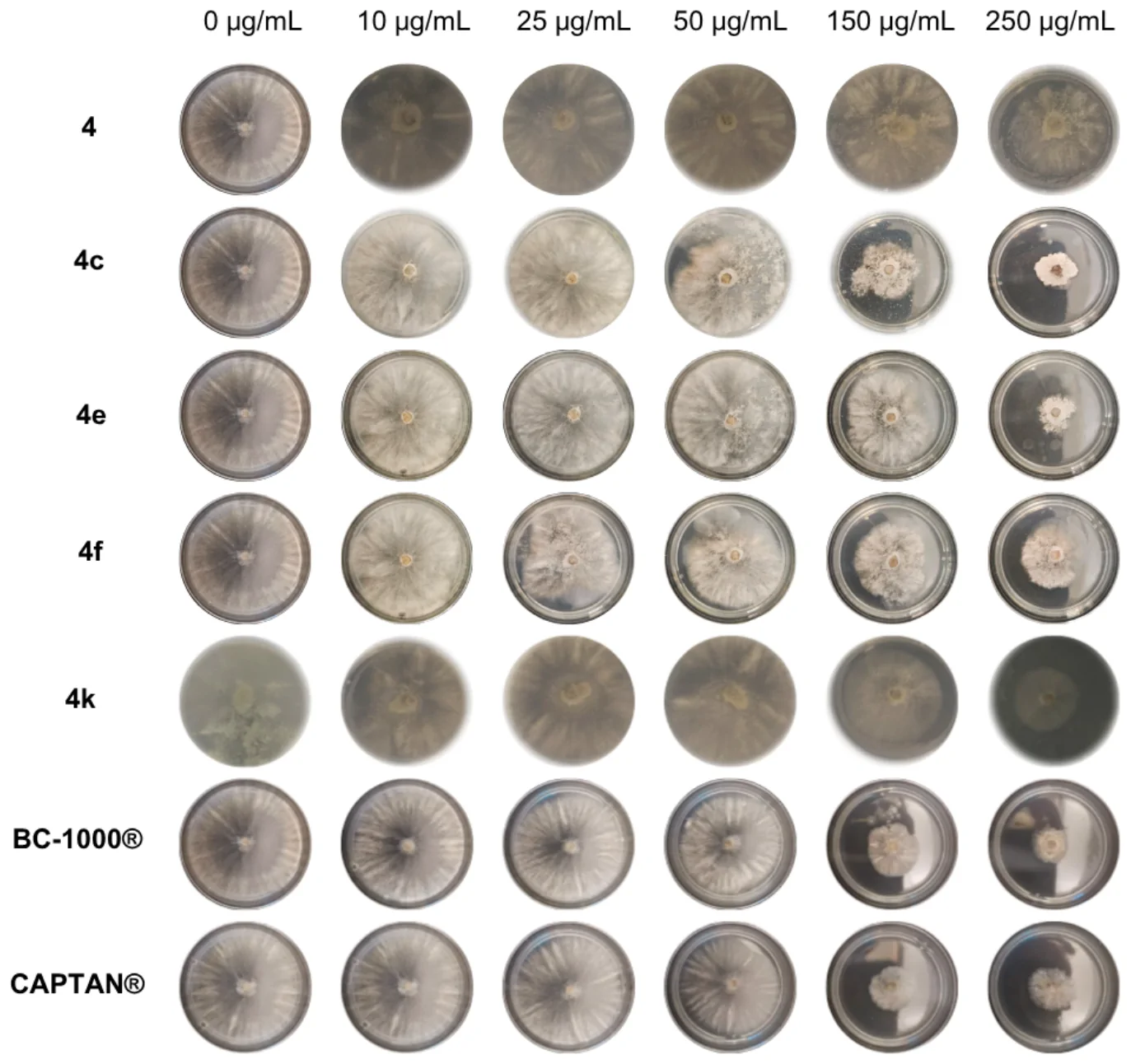
Concentration-dependent mycelial growth inhibition of *Botrytis cinerea* on semi-solid PDA medium treated with active 4-bromoindole derivatives (**4c**, **4e**, **4f**, and **4k**) and starting precursor (**4**) across concentrations ranging from 0 to 250 µg/mL.

When evaluated against *B. cinerea*, the unmodified starting core 4-bromoindole (**4**) showed complete inactivity (EC_50_ > 250 µg/mL). However, C3-acylation with specific aliphatic and aromatic moieties successfully activated the scaffold against this necrotrophic pathogen. Radial growth bioassays revealed that the butyric derivative (**4c**), the isovaleric derivative (**4f**), the valeric derivative (**4e**), and the 4-chlorobenzoyl derivative (**4k**) successfully inhibited mycelial growth in a concentration-dependent manner, achieving EC_50_ values of 127.27 µg/mL, 168.88 µg/mL, 186.53 µg/mL, and 201.83 µg/mL, respectively (Figure 1). Among them, compound **4c** stood out not only for displaying the strongest hyphal suppression within the 4-series against *B. cinerea*, but also for achieving an outstanding 98% conidial germination inhibition.

**Figure 2:**
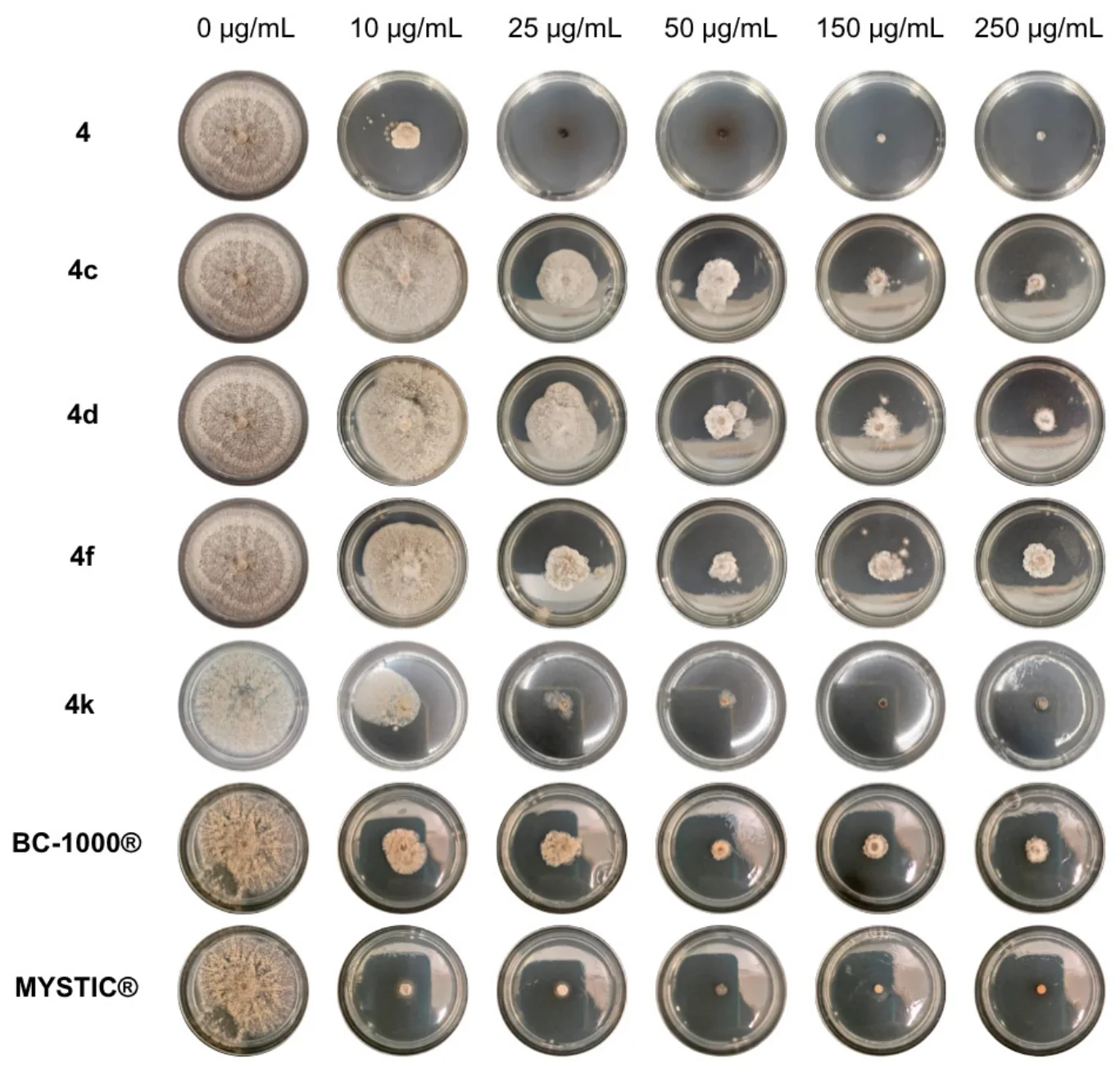
Concentration-dependent mycelial growth inhibition of *Monilinia fructicola* on semi-solid PDA medium treated with starting precursor (**4**) and active 4-bromoindole derivatives (**4c**, **4d**, **4f**, and **4k**) across concentrations ranging from 0 to 250 µg/mL.

In sharp contrast to the profile observed for *B. cinerea*, the susceptibility landscape changed drastically when the series was evaluated against *M. fructicola*. Here, the unmodified precursor 4-bromoindole (**4**) proved to be notably potent against vegetative mycelium, exhibiting an EC_50_ of 7.05 µg/mL (Figure 2). C3-acylation with halogenated aromatic functionalization preserved this high mycelial suppression, as evidenced by **4k** (EC_50_ = 12.21 µg/mL). Furthermore, aliphatic C3-modifications generated a robust active subgroup comprising **4f**, **4c**, and **4d** derivatives, which exhibited potent EC_50_ values of 16.74 µg/mL, 25.00 µg/mL, and 30.81 µg/mL, respectively. Notably, while parent compound **4** and derivative **4k** were completely incapable of blocking spore germination (0% ICG), the butyric derivative (**4c**) demonstrated dual-action efficacy, suppressing *M. fructicola* spore germination by 86%.

A comprehensive discussion of these data alongside previously published results for the 5-bromoindole (**5a**–**k**) [11] and 6-bromoindole (**6a**–**k**) [10] series demonstrates that the position of the bromine atom on the indole core governs pathogen selectivity and functional switching. In their unacylated core forms, C5 (EC_50_ = 12.17 µg/mL) [11] and C6 precursors (EC_50_ = 11.62 µg/mL) [10] act as potent mycelial growth inhibitors against *B. cinerea*, whereas the C4 precursor 4 is completely inactive against this pathogen (EC_50_ > 250 µg/mL). However, this selectivity reverses completely against *M. fructicola*, where precursor 4 exhibits superior baseline activity (EC_50_ = 7.05 µg/mL) compared to 5-bromoindole (7.91 µg/mL) [11] and 6-bromoindole (18.84 µg/mL) [10]. The introduction of a C3 acyl side chain acts as a key regulator of life-stage specificity: in Series 4, the four-carbon linear acyl chain in 3-butyryl-4-bromoindole (**4c**) provides the ideal steric and lipophilic balance, representing a promising dual-action candidate of the study by achieving simultaneous mycelial growth suppression and high spore germination inhibition against both *B. cinerea* (127.27 µg/mL; 98% ICG) and *M. fructicola* (25.00 µg/mL; 86% ICG); in Series 5, C3-acylation generally decreases mycelial inhibition, but aromatic modifications such as 4-chlorobenzoyl (**5k**) and 4-methoxybenzoyl (**5i**) redirect activity toward spore protection (81% and 63% ICG, respectively) [11]; whereas in Series 6, short-chain acetylation (**6a**) shifts the response toward preventive action (100% ICG on *B. cinerea* and 96% on *M. fructicola*), though hyphal growth inhibition drops significantly [10]. Overall, these structure–activity relationships establish that while halogenation at C5 or C6 favors curative mycelial disruption [10,11], C3-acylation with a four-carbon aliphatic chain (**4c**) on the C4-brominated scaffold provides an optimal balance, conferring dual curative–preventative potency across both postharvest fungal pathogens.

### 2.3. In Vivo Antifungal Efficacy Against Monilinia fructicola on Sweet Cherries

**Figure 3:**
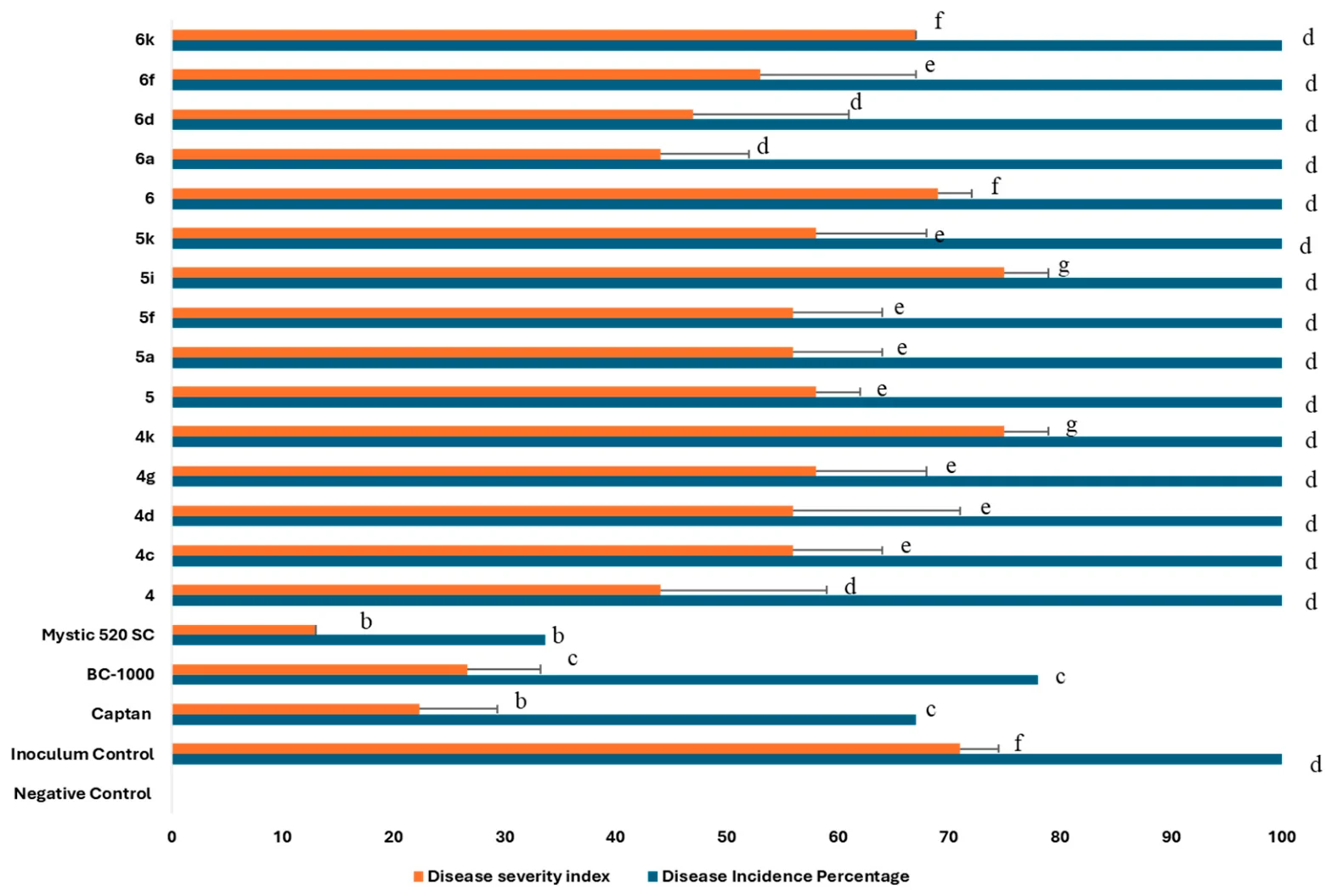
Effects of different treatments on the disease severity of Brown Rot caused by *Monilinia fructicola* in sweet cherries 5 days’ post-inoculation. Values represent mean disease severity indices. Disease severity data were analyzed using the Kruskal–Wallis test, followed by Dunn’s multiple-comparison test for pairwise comparisons. Differences were considered statistically significant at *p* < 0.05. Different letters indicate statistically significant differences among treatments.

**Figure 4:**
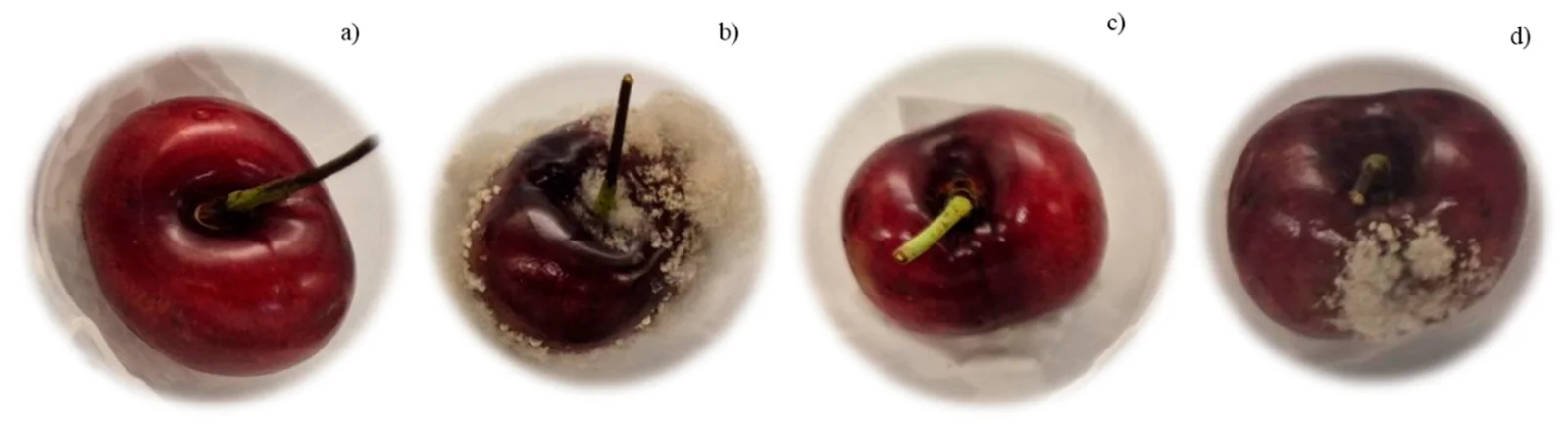
Representative visual symptoms of Brown Rot disease severity in sweet cherries wound-inoculated with *Monilinia fructicola* after 5 days of treatment: (**a**) uninoculated negative control (water only); (**b**) inoculated positive control (no fungicide applied); (**c**) commercial control (Mystic^®^ 520 SC at 0.4 mL/L); and (**d**) treatment with 4-bromoindole (**4**) at 250 mg/L.

To further validate the protective potential of the synthesized bromoindole derivatives under postharvest conditions, in vivo bioassays were conducted on sweet cherry fruits (*Prunus avium* L., cv. Lapins) wound-inoculated with *M. fructicola* (Figure 3 and Figure 4). *M. fructicola* was specifically selected for the fruit model due to the markedly higher susceptibility and superior inhibitory activity observed across the series during in vitro screening compared to *B. cinerea*. Representative top-performing candidates were selected from across the three regioisomeric series (4-, 5-, and 6-bromoindoles) to evaluate their preventive efficacy against Brown Rot development. Disease severity was evaluated 5 days post-inoculation to assess the performance of these selected compounds relative to commercial fungicides and control treatments (Assay results are in the Supplementary Material File S2).

Statistically significant differences in disease severity were observed across treatments (*p* < 0.05). The inoculated, untreated control fruit exhibited a high mean disease severity index of 71%, confirming active infection and rapid pathogen colonization under the assay conditions. The commercial synthetic benchmark Mystic^®^ 520 SC provided the highest level of fruit protection, suppressing disease severity down to 13%, followed by Captan^®^ (20%) and the organic formulation BC-1000^®^ (26.7%). Although the synthesized bromoindoles did not outperform the synthetic commercial fungicides, specific derivatives demonstrated significant postharvest protective capability compared to the untreated pathogen control. Among the tested molecules, 4-bromoindole (**4**), 3-acetyl-6-bromoindole (**6a**), and 3-butyryl-6-bromoindole (**6d**) emerged as the most effective candidates, maintaining disease severity values between 44% and 47%. This corresponds to an approximate 20% to 27% reduction in disease severity relative to the inoculated control, successfully delaying hyphal expansion and lesion spread in the fruit tissue.

Although compounds such as **4** and C3-acylated derivatives (e.g., **4c**) displayed outstanding in vitro intrinsic potency (EC_50_ = 7.05–25.00 µg/mL and up to 98% conidial germination inhibition), their in vivo performance on fruit tissue was more moderate, suppressing Brown Rot disease severity to 44–47%. This discrepancy between in vitro agar plate assays and in vivo fruit surface bioassays is a well-documented phenomenon in postharvest agrochemical research [31]. In culture media (PDA), lipophilic molecules are homogeneously dispersed using organic co-solvents (DMSO/ethanol), allowing direct, unhindered contact with fungal structures [32]; in contrast, on the cherry epicuticular surface and wounded mesocarp, several physicochemical barriers constrain compound bioavailability [33]. Bromoindoles possess high hydrophobic character, leading to poor dissolution in pure aqueous treatment baths and precipitation upon dilution, which limits effective molecular diffusion into the infection site [34,35]. Furthermore, the lipophilic nature of halogenated indoles causes preferential partitioning into the outer epicuticular wax layer of the fruit rather than penetrating deeper into wounded mesocarp tissue where *M. fructicola* hyphae proliferate [8,36], while unformulated active ingredients remain susceptible to physical displacement or rapid degradation [37]. To overcome these physical bottlenecks and unlock the full therapeutic potential of these bromoindoles in vivo, modern micro- and nano-formulation strategies represent a compelling solution [38]. Recent advancements demonstrate that encapsulating hydrophobic antifungal compounds into polymeric nanoparticles (e.g., chitosan), cyclodextrin inclusion complexes, nanoemulsions, or edible composite coatings (e.g., alginate) dramatically enhances aqueous dispersibility, protects active molecules from degradation, and ensures sustained controlled release at fruit wound sites [39,40]. Such nanoencapsulation systems increase localized tissue penetration and retention, bridging the gap between high in vitro intrinsic toxicity and practical in vivo disease suppression on harvested commodities [41]. Finally, it is important to clarify that the “sustainable” and “eco-friendly” claims made in this work refer strictly to the synthetic protocol and not to the toxicological profile of the final compounds. Although these derivatives showed promising in vivo antifungal activity, future cytotoxicity, selectivity index, and ecotoxicological studies are required before considering their practical application on edible crops. Driven by these findings, and in order to rationalize the intrinsic molecular binding affinities of these regioisomers independently of physical fruit matrix limitations, in silico molecular docking simulations were subsequently conducted against key fungal enzymatic targets.

### 2.4. In Silico Results

#### 2.4.1. Hansch Analysis and Univariate Examination of Antifungal Activity

**Figure 5:**
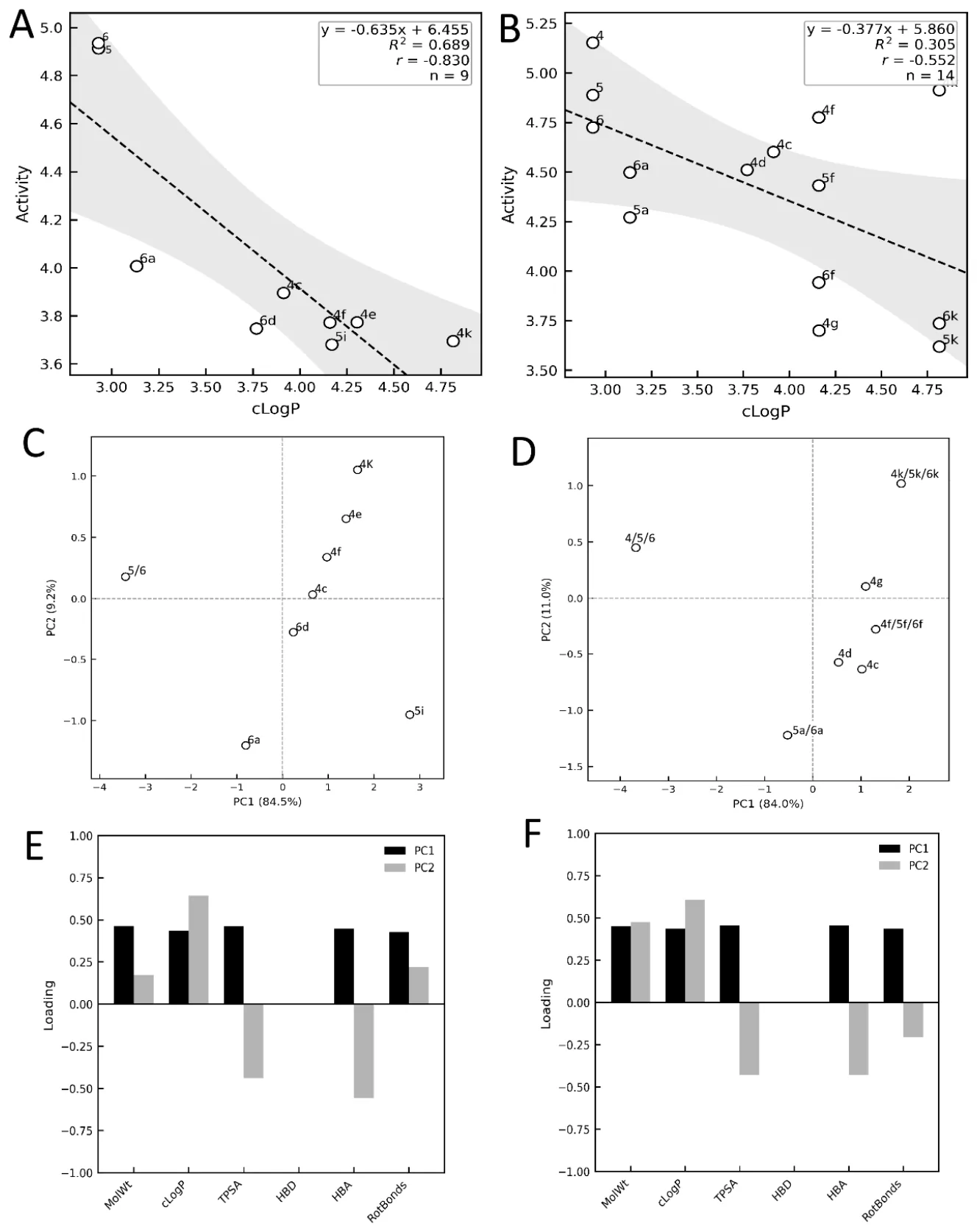
(**A**,**B**) Relationship between pEC50 and cLogP for *B. cinerea* and *M. fructicola*, respectively; (**C**,**D**) PC1 versus PC2 score plots for *B. cinerea* and *M. fructicola*. (**E**,**F**) Loading plots for *B. cinerea* and *M. fructicola*, respectively.

The exploratory Hansch analysis for *B. cinerea*, which examined the relationship between antifungal activity (pEC_50_) and lipophilicity (cLogP), revealed a moderate inverse linear association, with a coefficient of determination of R^2^ = 0.689 for a set of nine compounds. The calculated values are summarized in Table S1. Although the small dataset limits the development of statistically robust predictive models, the observed trend suggests that greater lipophilicity is associated with lower biological activity within this series (Figure 5A). In this context, lipophilicity appears to be a relevant descriptor that explains part of the variation in antifungal activity against *B. cinerea*, although it is clearly not the only factor involved. Internal leave-one-out (LOO) validation further showed that this single-descriptor model has limited robustness (*Q*^2^*_LOO_* = 0.395), supporting its exploratory rather than predictive nature.

For *M. fructicola*, the Hansch-type analysis also indicated an inverse relationship between pEC_50_ and cLogP, although the association was substantially weaker. Using the expanded dataset (n = 14), the linear fit yielded R^2^ = 0.305, with a moderate negative correlation (r = −0.552) and *p* = 0.0406. The calculated values are summarized in Table S2. The corresponding equation was Activity = −0.377·cLogP + 5.860 (Figure 5B). These findings indicate that lipophilicity alone does not adequately explain activity in this second series. This interpretation is reinforced by the LOO validation, which showed essentially no predictive stability for the univariate model (*Q^2^_LOO_* = 0.004). Therefore, although an inverse trend remains observable, its statistical and mechanistic significance should be interpreted with caution.

Taken together, these linear fits indicate that lipophilicity alone does not fully determine antifungal activity in either series. Rather, cLogP may represent a partial descriptor whose relevance is more evident for *B. cinerea* than for *M. fructicola*. In both cases, compounds with higher cLogP values tend to cluster within the lower pEC_50_ range, although the strength and stability of this trend are clearly target-dependent.

#### 2.4.2. Principal Component Analysis (PCA) for Multivariate Analysis

A principal component analysis (PCA) was performed to evaluate the simultaneous influence of multiple physicochemical properties on antifungal activity, using descriptors including molecular weight (MolWt), lipophilicity (cLogP), topological polar surface area (TPSA), hydrogen bond donors (HBD), hydrogen bond acceptors (HBA), and the number of rotatable bonds (RotBonds).

For *B. cinerea*, PC1 explained 84.5% of the variance, whereas PC2 accounted for 9.2%. The predominance of PC1 suggests that most descriptors covary within this dataset, consistent with substantial collinearity and with a compound set distributed mainly along a single overall physicochemical gradient. In this series, variation related to activity appears to be organized primarily along one dominant structural axis.

For *M. fructicola*, PCA of the updated dataset showed that PC1 explained 84.0% of the total variance, while PC2 explained 11.0%, giving a cumulative variance of 94.95% for the first two components. As in the *B. cinerea* series, structural variability appears to be captured largely by the first principal component. A further notable feature of this dataset is the recurrence of nearly identical PCA scores across several subsets of analogues, indicating that compounds with closely related substitution patterns also share highly similar overall descriptor profiles. In practical terms, this suggests that the descriptor space appears to be organized by broad physicochemical similarity. As in the first series, HBD was constant and therefore did not contribute meaningful information to the PCA.

Overall, the results provide a partially consistent picture for both fungal species. Figure 5A,B show negative slopes in the Hansch-type correlations, indicating that greater lipophilicity tends to be associated with lower activity in both systems. However, the strength of this trend differs substantially between the two series, and only in *B. cinerea* does the multivariate analysis suggest a relatively stable descriptor–activity relationship. The PCA score plots (Figure 5C,D) and loading plots (Figure 5E,F) refine this interpretation by showing that, in both cases, PC1 is dominated by global properties such as MolWt, cLogP, TPSA, HBA, and RotBonds. By contrast, PC2 reflects only secondary variation in the balance between lipophilicity and polarity-related features.

Accordingly, lower PC1 values are associated with higher activity in both datasets at the level of an apparent trend, suggesting that compounds located toward the lower end of the overall physicochemical gradient tend to be more active. However, this interpretation is supported much more strongly in the *B. cinerea* series than in *M. fructicola*. In the latter, the same physicochemical organization is present, but it does not translate into a comparably stable descriptor–activity model. Thus, although PCA suggests that both series are characterized by a similar underlying descriptor structure, only one displays a multivariate trend that remains meaningful after internal validation. Taken together, the Hansch and PCA/loading analyses suggest that antifungal activity in these series is influenced, at least in part, by global physicochemical properties rather than by any single descriptor considered in isolation. At the same time, the present results show that the extent to which these properties explain activity is target-dependent. For *B. cinerea*, a coherent multivariate pattern emerges; for *M. fructicola*, similar descriptors appear insufficient to capture the determinants of activity with comparable reliability. This distinction is important and cautions against overgeneralizing from one system to the other.

#### 2.4.3. Relationship Between pEC_50_ and PCA

**Figure 6:**
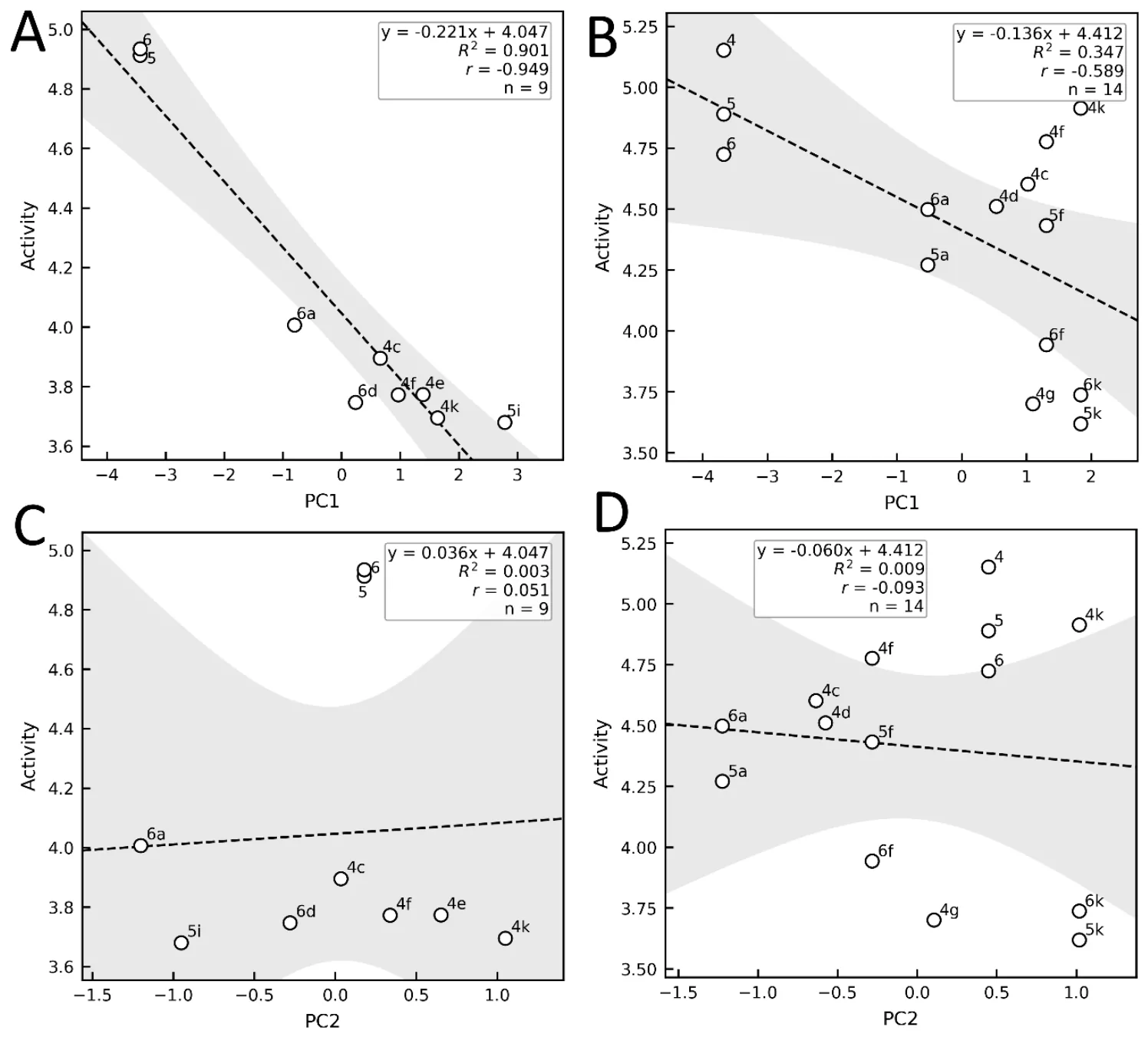
Relationship between pEC_50_ and PCA-derived descriptor axes. (**A**,**B**) Relationship of pEC_50_ with PC1 and PC2, respectively, for *B. cinerea*. (**C**,**D**) Relationship of pEC_50_ with PC1 and PC2, respectively, for *M. fructicola*.

Figure 6 shows a negative relationship between PC1 and activity against *B. cinerea* (Figure 6A,B), with a very strong linear fit for PC1 (R^2^ = 0.901), suggesting that a substantial proportion of the variation in pEC_50_ is associated with this principal component. In contrast, the relationship between activity and PC2 was negligible (R^2^ = 0.003), suggesting that this secondary axis captures little biologically relevant variation in this series. Importantly, the LOO comparison further supports this interpretation: whereas the single-descriptor Hansch model showed only moderate internal stability (*Q*^2^*_LOO_* = 0.395), the PCA-derived model based on PC1 retained substantial robustness (*Q*^2^*_LOO_* = 0.824). These results indicate that, for *B. cinerea*, the broader multivariate physicochemical profile represented by PC1 provides a more reliable description of activity than cLogP alone. For *M. fructicola* (Figure 6C,D), the relationship between activity and PC1 was also negative, although clearly weaker (R^2^ = 0.347 in the updated PCA–activity workflow; the LOO model comparison likewise indicated only a modest apparent fit), while the correlation between pEC_50_ and PC2 remained essentially null (R^2^ = 0.009). Most importantly, neither model showed satisfactory internal stability under LOO validation: *Q*^2^*_LOO_* was 0.004 for the Hansch model and only 0.147 for the PC1-based model. Accordingly, although PC1 represents the dominant descriptor axis of the series, its association with activity in *M. fructicola* should be regarded as exploratory rather than as evidence of a robust predictive relationship. These findings suggest that PC1 represents the principal descriptor axis associated with antifungal activity in both datasets at the level of apparent correlation, particularly against *B. cinerea*, whereas the variation captured by PC2 has negligible biological relevance in both systems. However, only the *B. cinerea* series supports the interpretation of a reasonably stable exploratory multivariate descriptor–activity relationship. In *M. fructicola*, the lack of internal robustness suggests that the current descriptor set does not adequately capture the factors governing activity and that additional descriptors or alternative structure-based approaches may be required. Overall, the updated analysis supports a more nuanced conclusion than the previous version. Both series appear to be organized around a dominant physicochemical gradient summarized by PC1, and in both cases PC2 is largely irrelevant with respect to activity. However, only in *B. cinerea* does this organization translate into a descriptor–activity relationship with appreciable internal consistency under LOO validation. Thus, although exploratory multivariate 2D-QSAR-like modeling appears informative for one fungal target, it remains insufficiently supported for the other.

#### 2.4.4. Molecular Docking Results with MfCat2 and SDH

**Figure 7:**
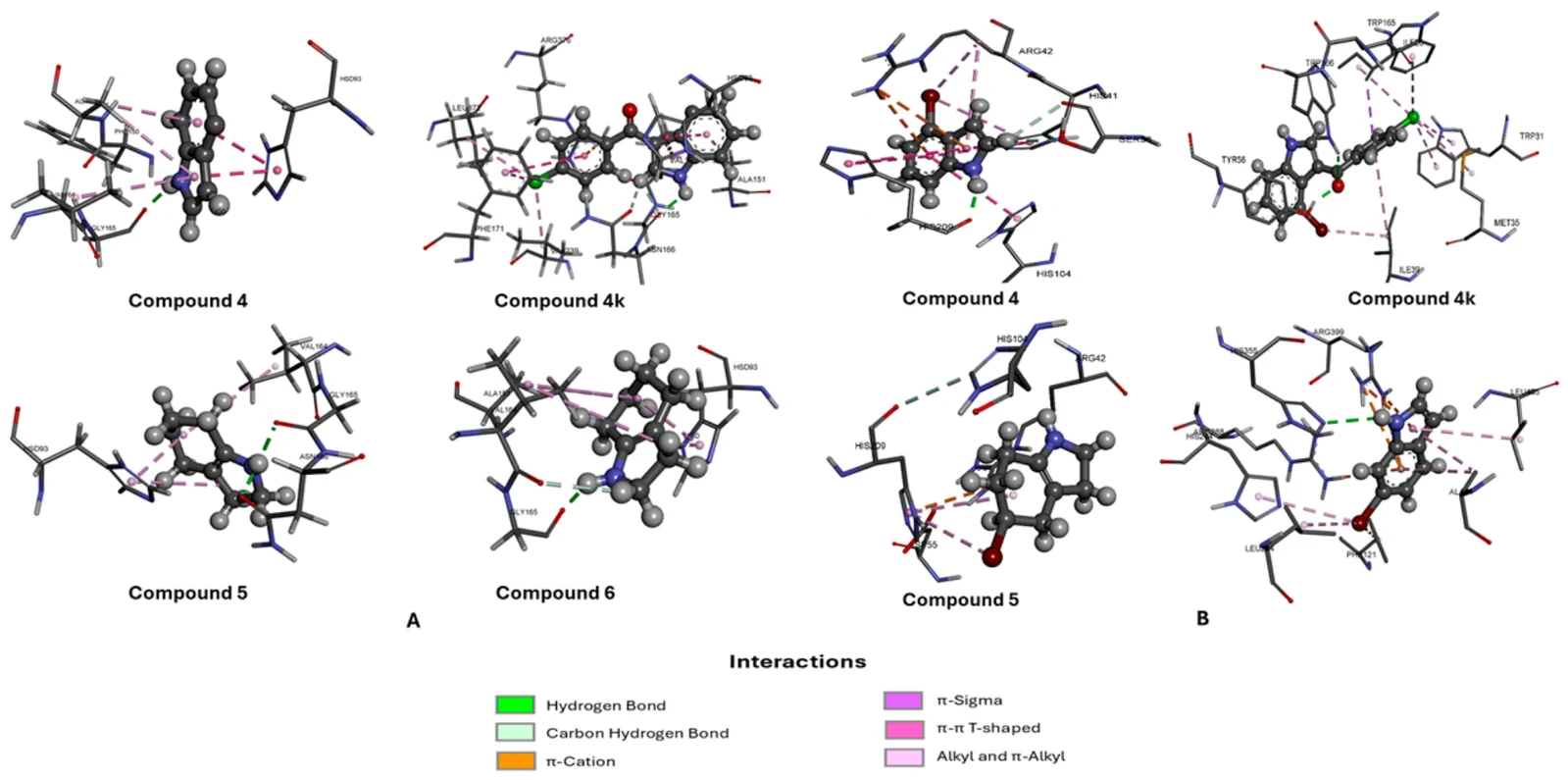
Three-dimensional representation of the predicted binding modes and key ligand–protein interactions of the selected bromoindole derivatives within the binding sites of MfCat2 (**A**) and SDH (**B**).

Molecular docking studies were performed to evaluate the binding affinity of the selected compounds **4**, **4k**, **5**, and **6** toward two relevant molecular targets in *M. fructicola*: catalase 2 (MfCat2) and succinate dehydrogenase (SDH). The predicted binding energies (ΔG, kcal/mol) and the main ligand–protein interactions are summarized in Table 2 and the interactions are shown in Figure 7.

Overall, all evaluated compounds showed favorable predicted binding energies toward both targets, suggesting their potential ability to interact with the binding pockets of MfCat2 and SDH. For MfCat2, compounds **5** and **6** exhibited the most favorable binding energies, with values of −8.96 and −8.78 kcal/mol, respectively, followed by **4k** (−7.82 kcal/mol) and **4** (−7.14 kcal/mol). These results are broadly compatible with the experimental activity profile, particularly for compounds **4**, **5**, and **6**, which were among the most active derivatives against *M. fructicola*. Compound **4k**, although showing slightly lower affinity than **5** and **6**, also displayed a favorable docking score, in agreement with its relevant in vitro activity. Analysis of the docking poses for MfCat2 revealed that all compounds established interactions with residues located in the same binding region, particularly Gly165, Hsd93, Val164, and Ala151, suggesting a potentially conserved binding mode. Compounds **4**, **4k**, and **6** formed hydrogen bonds with Gly165 at distances of 1.87, 1.87, and 1.82 Å, respectively, while compound 5 showed two hydrogen bonds involving Asn166 (2.07 Å) and Gly165 (2.02 Å). In addition, hydrophobic contacts with residues such as Val164, Ala151, Leu372, and Val239, as well as aromatic interactions involving Hsd93, contributed to ligand stabilization within the active site. For SDH, compound **6** exhibited the most favorable binding energy (−8.96 kcal/mol), followed by **4k** (−7.77 kcal/mol) and **4** (−7.68 kcal/mol), whereas compound **5** showed the least favorable affinity among the evaluated derivatives (−6.81 kcal/mol). These results suggest that, in contrast to MfCat2, SDH may be more selectively recognized by compounds **6**, **4k**, and **4**. Notably, compound **6** displayed the strongest predicted interaction with SDH, which is consistent with its balanced antifungal profile observed experimentally.

The interaction pattern within the SDH binding pocket differed among the compounds. Compound **4** formed a hydrogen bond with His209 (1.97 Å) and established additional contacts with His41, His104, Arg42, and Ser38. Compound **4k** showed a more diverse interaction profile, including hydrogen bonds with Tyr56 (1.86 Å) and Trp166 (2.73 Å), together with hydrophobic contacts involving Ile26, Ile39, Met35, Trp165, and Trp31. In contrast, compound **5** mainly established non-covalent contacts with Arg42, His209, Asp55, and His104, which is consistent with its comparatively lower docking score for this target. Compound **6** interacted favorably with SDH through a hydrogen bond with His355 (2.79 Å) and additional contacts with Arg288, Arg399, and Ala51, suggesting a favorable interaction pattern. Taken together, the docking results suggest that MfCat2 and SDH may represent plausible molecular targets for the selected 3-acyl-4-bromoindole derivatives. Although the predicted binding energies did not fully reproduce the experimental activity ranking, all compounds were predicted to establish favorable interactions within the active sites of both proteins, including hydrogen bonding and hydrophobic contacts with key residues. Notably, compounds **5** and **6** showed the most favorable affinity toward MfCat2, whereas compound **6** displayed the strongest interaction with SDH. These findings support the selection of compounds **4** and **6** for subsequent molecular dynamics simulations as representative ligands with relevant biological activity and favorable binding behavior. Although these docking results provide a plausible structural rationale for the observed antifungal activity, they should be regarded as predictive rather than definitive evidence of target engagement, and experimental validation will be required to confirm the proposed interactions.

#### 2.4.5. Molecular Dynamics Results

**Figure 8:**
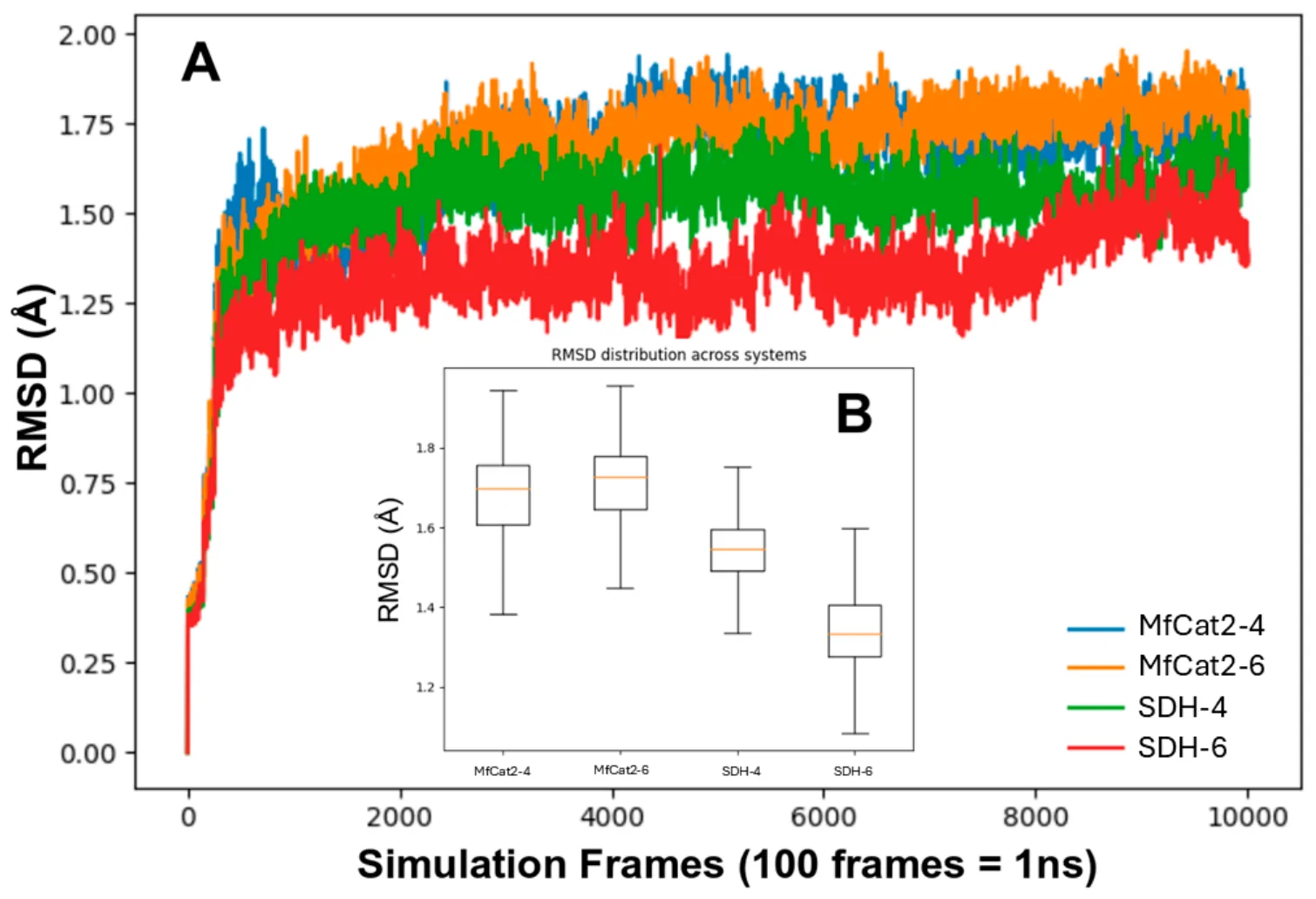
RMSD profiles of the studied protein–ligand complexes during 100 ns molecular dynamics simulations (**A**) and corresponding RMSD bar plot summary (**B**).

To further assess the stability of the selected ligand–protein complexes, 100 ns molecular dynamics simulations were performed for compounds **4** and **6** in complex with MfCat2 and SDH. In all systems, RMSD values remained below 2 Å, indicating overall structural stability throughout the simulations (Figure 8). Complexes involving MfCat2 showed slightly higher RMSD values than those involving SDH, suggesting a tendency toward greater conformational flexibility in the MfCat2 binding region under the simulated conditions.

**Figure 9:**
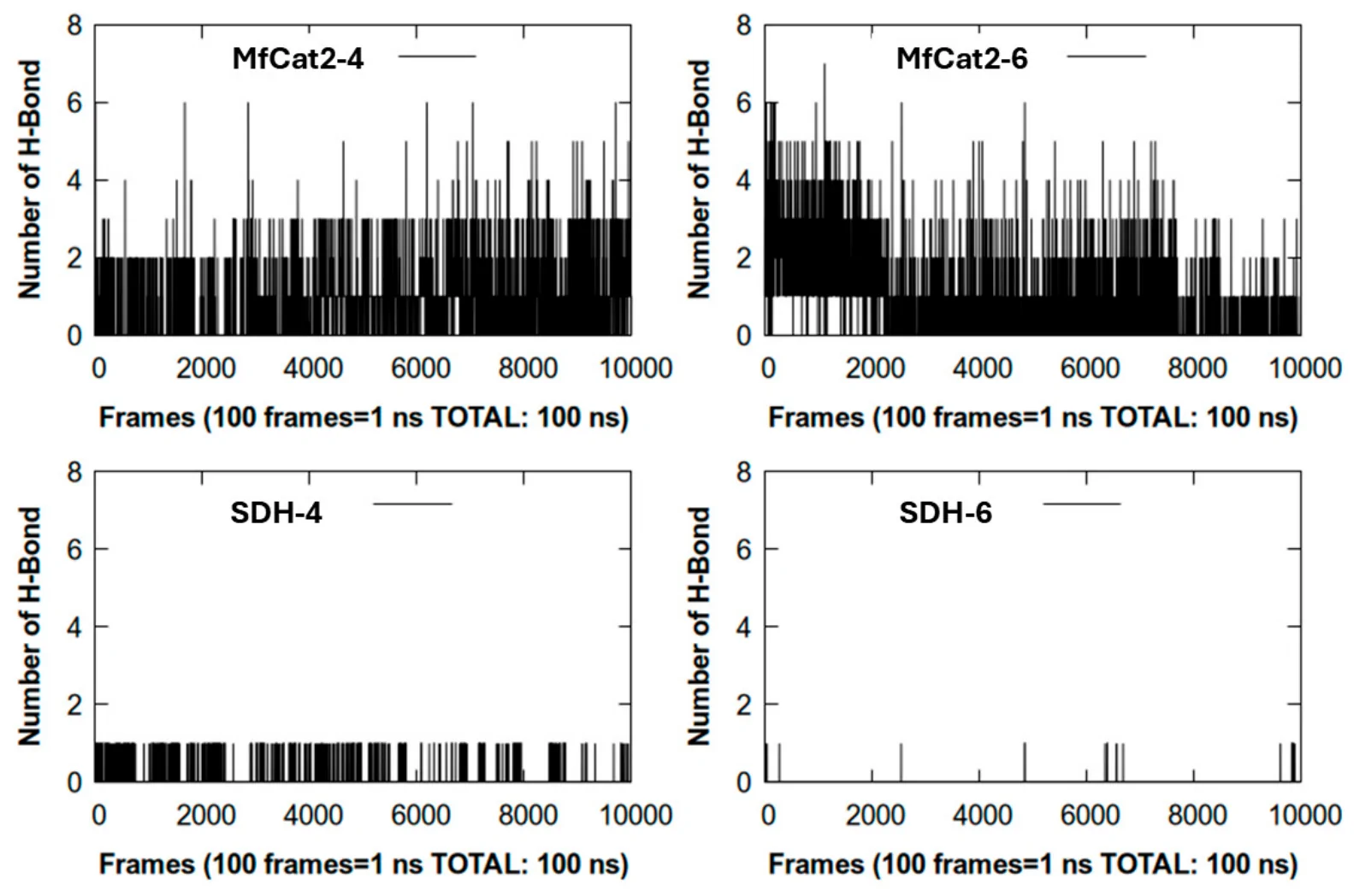
Hydrogen bond evolution during 100 ns of Molecular Dynamics simulations.

Analysis of hydrogen bond interactions (Figure 9) revealed a dynamic pattern of continuous formation and disruption along the trajectories. No hydrogen bond exceeded 50% occupancy, with the highest value corresponding to the interaction between Phe356 of MfCat2 and compound **4** (41.67%), indicating that these contacts were predominantly transient. A higher number of ligand–protein hydrogen bonds was observed for MfCat2 than for SDH, suggesting a greater propensity for transient polar interactions within the MfCat2 binding pocket during the simulations. Within the simulated trajectories, compound **4** exhibited a larger number of hydrogen bond interactions than compound **6**, consistent with its higher RMSD values and a more dynamic interaction profile.

**Figure 10:**
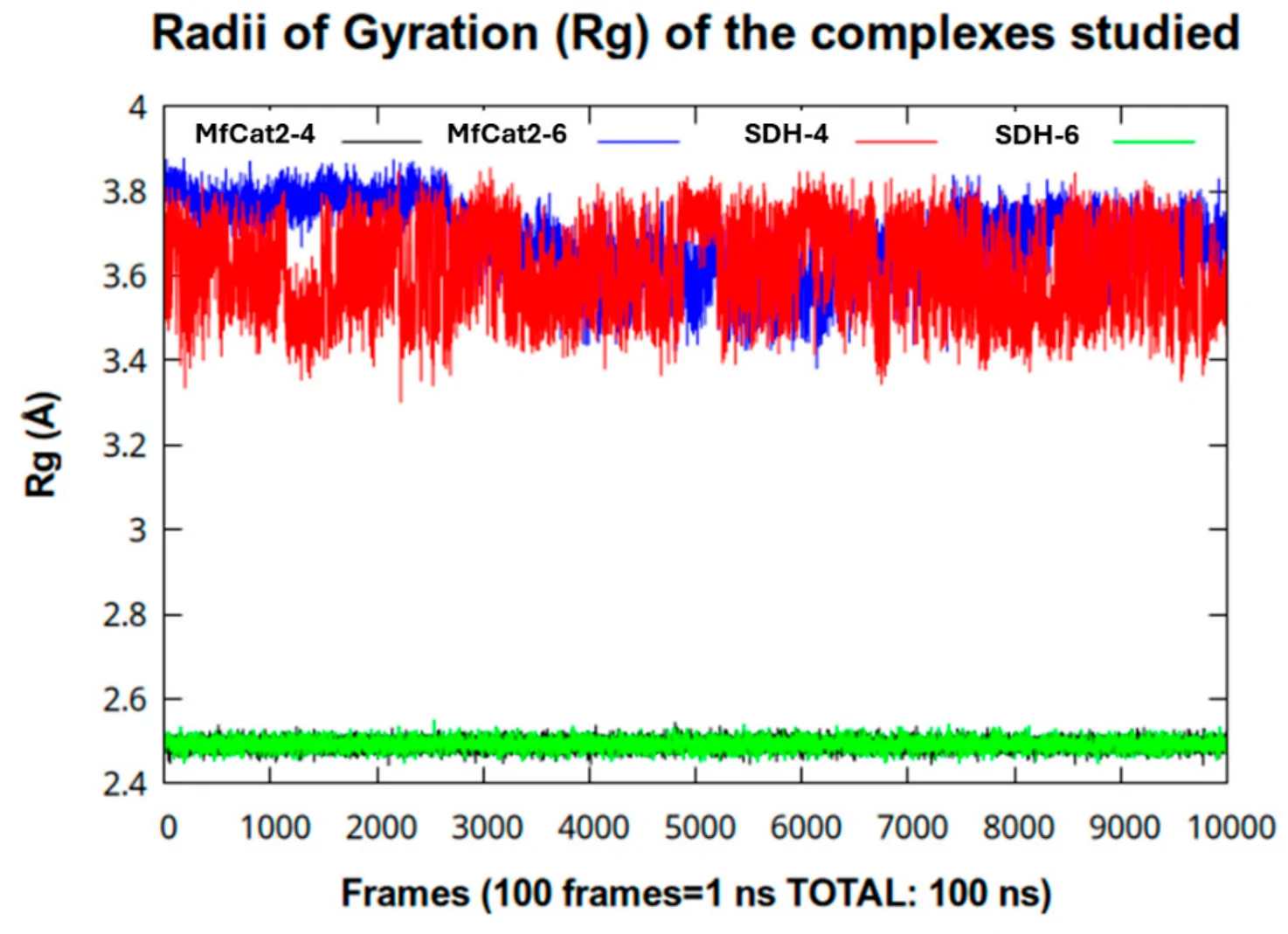
Radius of gyration (Rg) of the Cα atoms of the binding-site region in the studied protein-ligand complexes during 100 ns molecular dynamics simulations.

The radius of gyration (Rg) remained below 4 Å in all systems (Figure 10), indicating that the analyzed region preserved an overall compact conformation during the simulations. Slightly higher Rg values were observed for complexes with compound **6**, suggesting subtle differences in the interaction network within the binding site.

Overall, the MD simulations suggest that the selected ligand–protein complexes remained structurally stable throughout the simulated period and provide complementary mechanistic evidence regarding the dynamic behavior of compounds **4** and **6** within the predicted binding pockets of MfCat2 and SDH. These observations are consistent with, but do not demonstrate, the potential involvement of both proteins as molecular targets of the evaluated compounds. Taken together, the in silico studies performed in this work support succinate dehydrogenase (SDH) and catalase 2 from *M. fructicola* (MfCat2) as plausible molecular targets for the evaluated bromoindole derivatives. Although the docking scores did not fully reproduce the experimental ranking of antifungal activity, the selected compounds showed favorable binding energies and recurrent interactions with key residues in both proteins, supporting a multi-target mode of action rather than a strict affinity-activity correlation. Compounds **5** and **6** showed the most favorable binding energies toward MfCat2, whereas compound **6** displayed the strongest predicted affinity for SDH. These findings, together with the molecular dynamics simulations, provide complementary mechanistic evidence consistent with the potential biological relevance of both targets. These results are consistent with those reported by Silva et al. [11], who described favorable affinities of 3-acyl-5-bromoindole derivatives toward SDH and their association with antifungal activity against *M. fructicola* and *B. cinerea*. Likewise, in agreement with Muñoz et al. [10], the present study supports the hypothesis that structural modifications of the bromoindole scaffold may modulate target preference toward SDH or catalase-related enzymes such as MfCat2, reinforcing the proposal of a multi-target antifungal mechanism. In addition, the use of conserved structural models allowed the identification of ligand–protein interaction patterns comparable to those reported for other SDH-targeting fungicides [42,43]. Despite these promising results, the theoretical approaches employed in this study have inherent limitations. Molecular docking and molecular dynamics simulations are predictive computational approaches whose reliability depends on the quality of the protein structures employed, particularly when homology models are used, which may introduce uncertainties associated with species-specific structural differences. In addition, these methods do not account for important biological factors such as membrane permeability, intracellular accumulation, metabolism, or off-target effects. Therefore, the computational results should be regarded as exploratory mechanistic evidence that supports, but does not demonstrate, the proposed interactions between the evaluated compounds and the selected protein targets. Experimental validation will be required to confirm their molecular mechanism of action. Furthermore, although the 100 ns molecular dynamics simulations indicated stable binding modes throughout the simulation period, only a single trajectory was generated for each complex. Consequently, comparisons among compounds should be interpreted qualitatively rather than quantitatively. Independent replicate simulations would strengthen the statistical robustness and reproducibility of these observations.

## 3. Materials and Methods

### 3.1. General Information

All solvents, chemicals, and reagents, including bromoindoles, were purchased from AK Scientific Inc. (Union City, CA, USA) or Merck (Darmstadt, Germany) at the highest commercially available purity and used as received without further purification.

The synthesized compounds were isolated by column chromatography (CC) using Merck silica gel (200–300 mesh), and reaction progress was monitored by thin-layer chromatography (TLC) on silica gel HF-254 plates, visualized by spraying with 25% aqueous H_2_SO_4_ followed by heating. The structures of all products were determined by melting point, NMR, and mass spectrometry analysis. Melting points were measured on a Stuart Scientific SMP3 apparatus. NMR spectra (^1^H at 400.1 MHz, ^13^C at 100.6 MHz) were recorded on a Bruker Avance 400 spectrometer in CDCl_3_ or CD_3_COCD_3_. Chemical shifts (*δ*) are reported in ppm relative to residual solvent peaks (CDCl_3_: *δ*_H_ 7.26, δ_C_ 77.0; CD_3_COCD_3_: *δ*_H_ 2.04, δ_C_ 29.8), and coupling constants (*J*) are given in Hz. Mass spectra were acquired on a Shimadzu GCMS-QP5050A system or an Agilent 7890B GC coupled to a 5977A MS (EI, 70 eV) equipped with an HP-5MS column. Analysis used helium (1 mL/min), an oven ramp from 50 °C (1 min) to 320 °C (2 min) at 10 °C/min, and 1 µL injections (1 mg/mL in MeCN, split 1:50) in SCAN mode via Agilent MassHunter v10.0.

### 3.2. Synthesis of 3-Acyl-bromoindoles (**4a**–**k**, **5a**–**k**, and **6a**–**k**)

The target molecules were synthesized in a CEM Discover 2.0 microwave synthesizer (CEM Corporation, Matthews, NC, USA) by adapting and optimizing a previously reported method [10,11,28]. In a typical procedure, a 10 mL microwave pressure vessel was charged with the corresponding bromoindole regioisomer (4-, 5-, or 6-bromoindole, 1.0 mmol), the appropriate acid anhydride (linear or aromatic, 1.0 mmol), Y(OTf)_3_, 0.01 mmol as catalyst, and the ionic liquid [BMI]BF_4_ (1.0 mmol) as the reaction medium. The vessel was sealed with a Teflon/silicone septum and irradiated under specific temperature, time, and power settings according to the nature of the acylating agent. Specifically, reactions with linear or branched aliphatic anhydrides (Condition A, yielding derivatives **4a**–**f**, **5a**–**f**, and **6a**–**f**) were conducted at 70–90 °C for 5–15 min with the power output set to 100 W, whereas reactions with aromatic anhydrides (Condition B, yielding derivatives **4g**–**k**, **5g**–**k**, and **6g**–**k**) were performed at 90–120 °C for 20–40 min at 150 W (the exact optimal temperature and irradiation time for each individual derivative are summarized in Table S3 in the Supplementary Materials). Upon completion, the mixture was rapidly cooled to 50 °C using the integrated compressed air system and then allowed to reach room temperature. The crude mixture was extracted with Et_2_O (5 × 10 mL), and the combined organic layers were washed with water (2 × 10 mL), saturated aqueous NaHCO_3_ (2 × 20 mL), and brine (2 × 10 mL). The organic phase was dried over anhydrous MgSO_4_, filtered through Whatman No. 1 paper, and concentrated under reduced pressure using a rotary evaporator. Finally, aliphatic derivatives (**4a**–**4f**) were purified by recrystallization from ethyl ether/hexane (1:3, *v*/*v*), whereas aromatic derivatives (**4g**–**k**) were purified by flash column chromatography on silica gel using hexane/ethyl acetate (8:2, *v*/*v*) as the eluent to determine the isolated yields.

The known linear acylated derivatives 1-(4-bromo-1H-indol-3-yl)ethan-1-one (**4a**) 1-(4-bromo-1H-indol-3-yl)propan-1-one (**4b**) and 1-(4-bromo-1H-indol-3-yl)butan-1-one (**4c**) were isolated as a light brown solid (68.7% yield; m.p.: 180–181 °C), a white solid (68.8% yield; m.p.: 154–155 °C), and a white solid (63.5% yield; m.p.: 154–155 °C), respectively. Spectroscopic data of compounds **4a**-**4c** were consistent with those reported in the literature [24,26]. The new linear acylated derivatives are described below (see Supplementary Material File S1):

1-(4-bromo-1H-indol-3-yl)-2-methylpropan-1-one (**4d**): The compound was isolated as a white solid with a yield of 55.0%. m.p.: 104–105 °C. ^1^H NMR (400 MHz, CD_3_COCD_3_): δ 8.10 (s, 1H, H-2); 7.52 (d, *J* = 8.1 Hz, 1H, H-7); 7.38 (d, *J* = 7.6 Hz, 1H, H-5); 7.13–7.09 (m, 1H, H-6); 3.43–3.37 (m, 1H, H-2′); 1.16 (d, *J* = 6.8 Hz, 6H, H-3′ and H-4′). ^13^C NMR (100 MHz, CD_3_COCD_3_): δ 201.8 (C-1′); 138.0 (C-8); 130.2 (C-2); 126.9 (C-9); 124.9 (C-6); 124.2 (C-5); 118.5 (C-3); 114.5 (C-4); 110.0 (C-7); 40.1 (C-2′); 19.4 (C-3′ and C-4′). EI-MS, *m*/*z* (%): 267 (8), 265 (8), 224 (94), 222 (100), 194 (12), 143 (15), 115 (24).

1-(4-bromo-1H-indol-3-yl)pentan-1-one (**4e**): The compound was isolated as a white solid with a yield of 80.6%. m.p.: 101–102 °C. ^1^H NMR (400 MHz, CD_3_COCD_3_): δ 8.20 (s, 1H, H-2); 7.51 (d, *J* = 8.1 Hz, 1H, H-7); 7.39 (d, *J* = 7.6 Hz, 1H, H-5); 7.12–7.08 (m, 1H, H-6); 2.89 (t, *J* = 7.3 Hz, 2H, H-2′); 1.72–1.65 (m, 2H, H-3′); 1.45–1.35 (m, 2H, H-4′); 0.92 (t, *J* = 7.4 Hz, 3H, H-5′). ^13^C NMR (100 MHz, CD_3_COCD_3_): δ 195.1 (C-1′); 139.5 (C-8); 133.2 (C-2); 127.4 (C-9); 125.7 (C-6); 124.6 (C-5); 119.8 (C-3); 115.0 (C-4); 112.1 (C-7); 42.2 (C-2′); 28.0 (C-3′); 23.0 (C-4′); 14.3 (C-5′). EI-MS, *m*/*z* (%): 281 (8), 279 (10), 237 (36), 224 (90), 222 (100), 194 (12), 143 (12), 115 (26).

1-(4-bromo-1H-indol-3-yl)-3-methylbutan-1-one (**4f**): The compound was isolated as a white solid with a yield of 53.2%. m.p.: 130–131 °C. ^1^H NMR (400 MHz, CD_3_COCD_3_): δ 8.20 (s, 1H, H-2); 7.51 (d, *J* = 8.1 Hz, 1H, H-7); 7.39 (d, *J* = 7.6 Hz, 1H, H-5); 7.13–7.09 (m, 1H, H-6); 2.76 (d, *J* = 7.0 Hz, 2H, H-2′); 2.28–2.18 (m, 1H, H-3′); 0.98 (d, *J* = 6.7 Hz, 6H, H-4′ and H-5′). ^13^C NMR (100 MHz, CD_3_COCD_3_): δ 196.8 (C-1′); 138.2 (C-8); 131.5 (C-2); 127.3 (C-9); 124.6 (C-6); 124.3 (C-5); 119.7 (C-3); 114.6 (C-4); 111.0 (C-7); 51.4 (C-2′); 26.1 (C-3′); 22.7 (C-4′ and C-5′). EI-MS, *m*/*z* (%): 281 (14), 280 (4) M^+^, 279 (14), 265 (6), 237 (15), 224 (94), 222 (100), 196 (10), 143 (8), 115 (24).

The synthesis of the known acylated aromatic derivative (6-bromo-1H-indol-3-yl)(phenyl)methanone (**4g**) yielded a white solid (m.p.: 191–192 °C) in 53.2% yield. The spectroscopic data obtained for this derivative are consistent with those reported in previous studies [27,28,29]. The synthesis and characterization of the new derivatives are described below (see Supplementary Material File S1):

(4-bromo-1H-indol-3-yl)(4-methylphenyl)methanone (**4h**): The compound was isolated as a white solid with a yield of 51.6%. m.p.: 180–181 °C. ^1^H NMR (400 MHz, CD_3_COCD_3_): δ 7.79–7.76 (m, 3H, H-2, H-4′ and H-6′); 7.58 (d, *J* = 8.0 Hz, 1H, H-7); 7.36 (d, *J* = 7.7 Hz, 1H, H-5); 7.30 (d, 2H, *J* = 7.9 Hz, H-3′ and H-7′); 7.15 (t, *J* = 7.9 Hz, 1H, H-6); 2.41 (s, 3H, CH_3_). ^13^C NMR (100 MHz, CD_3_COCD_3_): δ 189.8 (C-1′); 143.5 (C-5′); 139.0 (C-2′); 138.7 (C-8); 133.0 (C-2); 130.6 (C-4′ and C-6′); 129.7 (C-3′ and C-7′); 126.7 (C-9); 126.6 (C-6); 124.6 (C-5); 118.3 (C-3); 114.9 (C-4); 112.3 (C-7); 21.5 (CH_3_Ar). EI-MS, *m*/*z* (%): 314 (5) M^+^, 234 (50), 224 (90), 222 (100), 143 (18), 115 (24).

(4-bromo-1H-indol-3-yl)(4-methoxyphenyl)methanone (**4i**): The compound was isolated as a white solid with a yield of 47.9%. m.p.: 196–197 °C. ^1^H NMR (400 MHz, CD_3_COCD_3_): δ 7.88–7.85 (m, 2H, H-4′ and H-6′); 7.75 (s, 1H, H-2); 7.58 (d, *J* = 8.0 Hz, 1H, H-7); 7.36 (d, *J* = 7.7 Hz, 1H, H-5); 7.15 (t, *J* = 7.9 Hz, 1H, H-6); 7.03–7.00 (m, 2H, H-3′ and H-7′); 3.88 (s, 3H, CH_3_O). ^13^C NMR (100 MHz, CD_3_COCD_3_): δ 188.8 (C-1′); 163.7 (C-5′); 138.6 (C-8); 133.8 (C-2′); 133.8 (C-2); 131.9 (C-3′ and C-7′); 126.4 (C-9); 126.1 (C-6); 124.2 (C-5); 118.1 (C-3); 114.6 (C-4); 114.0 (C-4′ and C-6′); 112.0 (C-7); 55.5 (CH_3_OAr). EI-MS, *m*/*z* (%): 330 (12) M^+^, 329 (70), 250 (80), 224 (92), 222 (100), 207 (35), 135 (42), 115 (28).

(4-bromo-1H-indol-3-yl)(4-fluorophenyl)methanone (**4j**): The compound was isolated as a white solid with a yield of 23.8%. m.p.: 220–221 °C. ^1^H NMR (400 MHz, CD_3_COCD_3_): δ 7.95 (m, 2H, H-4′ and H-6′); 7.83 (s, 1H, H-2); 7.60 (d, *J* = 8.2 Hz, 1H, H-7); 7.39 (d, *J* = 7.6 Hz, 1H, H-5); 7.29–7.23 (m, 2H, H-3′ and H-7′); 7.17 (t, *J* = 7.9 Hz, 1H, H-6). ^13^C NMR (100 MHz, CD_3_COCD_3_): δ 188.4 (C-1′); 165.7(C-5′); 138.6 (C-8); 137.6 (C-2′); 133.1 (C-2); 132.8 (C-3′ and C-7′); 126.4 (C-9); 126.3 (C-6); 124.5 (C-5); 117.7 (C-3); 115.7 (C-4′ and C-6′); 114.5 (C-4); 112.0 (C-7). EI-MS, *m*/*z* (%): 318 (8) M^+^, 317 (62), 238 (56), 224 (96), 222 (100), 182 (14), 143 (12), 115 (24), 95 (22).

(4-bromo-1H-indol-3-yl)(4-chlorophenyl)methanone (**4k**): The compound was isolated as a white solid with a yield of 20.9%. m.p.: 145–146 °C. ^1^H NMR (400 MHz, CD_3_COCD_3_): δ 7.85 (d, *J* = 8.5 Hz, 2H, H-4′ and H-6′); 7.54 (s, 1H, H-2); 7.45–7.41 (m, 4H, H-5, H-7, H-2′ and H-6′); 7.16 (t, *J* = 8.0 Hz, 1H, H-6). ^13^C NMR (100 MHz, CD_3_COCD_3_): δ 189.2 (C-1′); 138.9 (C-5′); 138.3 (C-8); 137.4 (C-2′); 133.8 (C-2); 131.3 (C-3′ and C-7′); 128.6 (C-4′ and C-6′); 126.4 (C-9); 125.6 (C-6); 124.6 (C-5); 118.2 (C-3); 114.8 (C-4); 110.8 (C-7). EI-MS, *m*/*z* (%): 335 (52) M^+^, 333 (40), 224 (92), 222 (100), 207 (24).

### 3.3. In Vitro Antifungal Efficacy Evaluation

#### 3.3.1. Fungal Isolates and Culture Conditions

The phytopathogenic fungi *Botrytis cinerea* (UK-1, isolated from *Vitis vinifera*) and *Monilinia fructicola* (S1, isolated from *Prunus persica*) were obtained from the culture collection of the Laboratorio de Pruebas Biológicas (Universidad Técnica Federico Santa María, Chile). Master strains were maintained on potato dextrose agar (PDA; Difco, Detroit, MI, USA) at 4 °C. To generate fresh inoculum for the biological evaluation, fungal isolates were grown on PDA plates at 23 °C for 5 days under a 16 h light/8 h dark photoperiod.

#### 3.3.2. In Vitro Radial Growth Assay

The antifungal efficacy of the 3-acyl-bromoindoles (**4a**–**k**, **5a**–**k**, and **6a**–**k**), along with three commercial reference fungicides (BC-1000^®^, Mystic^®^ 520 SC, and Captan^®^ 13W), was determined using a radial growth inhibition assay on PDA [44]. Test compounds were incorporated into molten PDA to achieve final concentrations ranging from 0 to 250 µg/mL. Control plates were supplemented with 1% (*v*/*v*) ethanol, matching the highest solvent content used. Each plate was centrally inoculated with a 4 mm mycelial plug. Inoculated cultures were kept in complete darkness at 23 °C for 3 days (*B. cinerea*) or 5 days (*M. fructicola*). All assays were performed in triplicate. Following incubation, radial growth diameters were recorded, and the percentage of mycelial inhibition was determined relative to the solvent control. Half-maximal effective concentrations (EC_50_, defined as the dose suppressing 50% of mycelial growth) were calculated via non-linear regression analysis of dose–response curves using OriginPro v.8 software. Statistical significance was evaluated using one-way analysis of variance (ANOVA) followed by Fisher’s LSD post hoc test (*p* < 0.05).

#### 3.3.3. Spore Germination Assay

Inhibition of conidial germination (ICG) was evaluated following a modified protocol [45]. Aliquots (40 µL) of a spore suspension (1 ×10^5^ conidia/mL), prepared from 6-day-old (*B. cinerea*) or 9-day-old (*M. fructicola*) cultures, were uniformly spread onto PDA plates amended with the 3-acyl-bromoindoles **4a**–**k**, **5a**–**k**, and **6a**–**k** (10–250 µg/mL). Following a 16 h dark incubation period at 23 °C, 100 conidia per replicate were microscopically scored to evaluate germination. A conidium was marked as germinated when its germ tube exceeded twice its length.

### 3.4. In Vivo Antifungal Evaluation

#### 3.4.1. Antifungal Efficacy on Sweet Cherry Fruit

**Figure 11:**
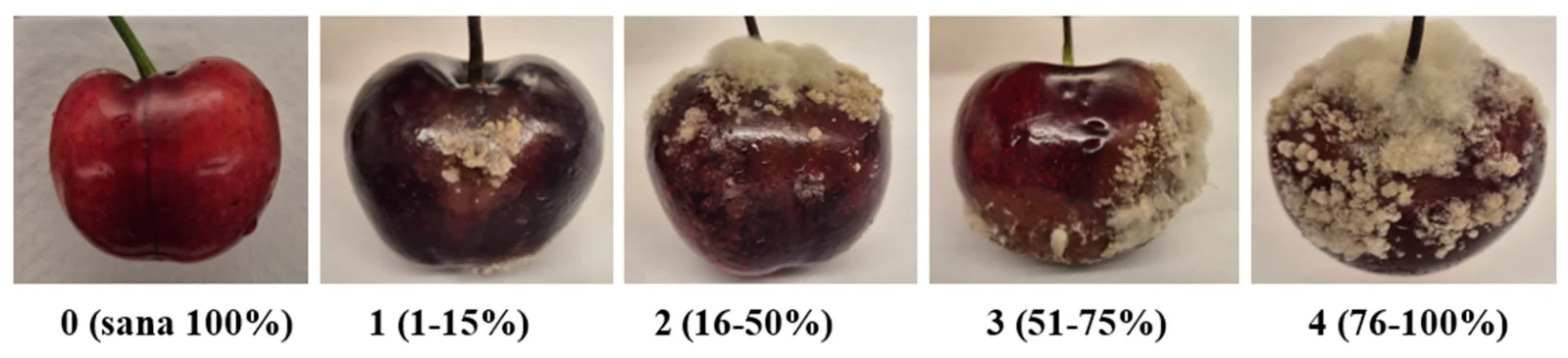
Visual rating scale (0–4) of symptoms caused by *M. fructicola* on sweet cherries 5 days post-inoculation: 0 = no signs of disease (uninfected, 100% healthy); 1 = 1–15% visible lesion without sporulation (mild infection); 2 = 16–50% sporulated area covering less than half of the fruit (moderate infection); 3 = 51–75% sporulated area covering more than half of the fruit (severe infection); 4 = 76–100% sporulated area covering the fruit surface (very severe infection).

Fresh sweet cherries (*Prunus avium* L. cv. Lapins) were harvested from the La Palma Experimental Station of the Pontifical Catholic University of Valparaíso (Quillota, Valparaíso Region, Chile). Fruits of uniform size and without mechanical damage or visible signs of infection were selected. The cherries were surface-sterilized for 2 min in a 1% (*v*/*v*) sodium hypochlorite (NaClO) solution, rinsed thrice with sterile distilled water, and dried in a laminar flow hood. Randomized groups of 10 fruits per treatment were placed into 10-compartment plastic containers lined with paper towels moistened with 100 µL of sterile water to maintain high relative humidity (90–95%). Two hours prior to treatment, each cherry was wounded at the equatorial region using a sterile needle (1 mm diameter). Based on the in vitro bioassay results, representative 3-acyl-bromoindoles from each series displaying high, moderate, and inactive antifungal profiles were evaluated at 250 mg/L. Reference commercial fungicides (Captan^®^, 1.5 mL/L; BC-1000^®^, 1.8 g/L; Mystic^®^ 520 SC, 0.4 mL/L) were tested under the same conditions. All treatments were applied by spraying onto the fruit surface. Subsequently, 20 µL of an *M. fructicola* conidial suspension (1×10^5^ conidia/mL) was injected into each wound. Inoculated cherries sprayed with sterile water served as the positive control (disease control), whereas uninoculated and untreated fruits served as the negative control to evaluate baseline fruit quality. Treatments were incubated at 24 °C for 5 days post-inoculation. Disease incidence (% of infected fruit) and the disease severity index (DSI) were recorded after the incubation period. Lesion severity was evaluated using a 0-to-4 rating scale (Figure 11), and the DSI was calculated according to established methods [46]. To fulfill Koch’s postulates, *M. fructicola* identity was confirmed by re-isolation from the margins of active lesions (1.5 cm tissue samples) onto PDA plates.

#### 3.4.2. Statistical Analysis

Disease severity index data were analyzed using non-parametric statistical methods. Differences among treatments were assessed using the Kruskal–Wallis test, followed by Dunn’s post hoc test for multiple pairwise comparisons. Statistical significance was established at *p* < 0.05. Different letters indicate statistically significant differences among treatments.

### 3.5. In Silico Studies

#### 3.5.1. Dataset and Calculation of Physicochemical Descriptors

The dataset comprised a series of 3-acyl-bromoindoles evaluated for antifungal activity against *B. cinerea* and *M. fructicola*. Biological activity was expressed as EC_50_ values and transformed to negative logarithmic scale (pEC_50_ = –log_10_(EC_50_)) to linearize structure–activity relationships and to facilitate statistical analysis. Compounds with non-quantifiable activity (EC_50_ > 250 µg/mL) were excluded from the EC_50_ transformation and regression fitting due to the lack of a discrete numerical activity value.

Chemical structures were encoded as SMILES and converted into 3D molecular representations using the RDKit open-source cheminformatics toolkit in Python. For each derivative, an initial 3D conformation was generated via the ETKDG algorithm and subsequently energy-minimized using the Universal Force Field (UFF). From these optimized structures, a set of global physicochemical descriptors commonly employed in QSAR modeling was calculated: molecular weight (MW), calculated partition coefficient (cLogP), topological polar surface area (TPSA), number of hydrogen bond donors (HBD) and acceptors (HBA), and number of rotatable bonds (RotBonds).

#### 3.5.2. Exploratory Hansch-Type QSAR Analysis

An exploratory quantitative structure–activity relationship (QSAR) analysis was conducted to evaluate the correlation between antifungal potency and the calculated physicochemical descriptors. Given the dataset size, a classic Hansch-type approach based on ordinary least squares linear regression was employed, selecting lipophilicity (cLogP) as the primary descriptor against pEC_50_ values. Linear regressions and associated statistical metrics, including the coefficient of determination (R^2^), standard error of the estimate, and sample size (n), were computed using the scikit-learn package in Python. These parameters were used strictly for descriptive and interpretative purposes rather than for predictive modeling.

#### 3.5.3. Principal Component Analysis

Principal component analysis (PCA) was performed to explore the physicochemical space of the synthesized compounds in a multivariate context and to complement the univariate QSAR evaluation. Prior to analysis, all calculated physicochemical descriptors were mean-centered and auto-scaled to unit variance to prevent bias resulting from differences in units or scale magnitudes among variables. PCA was computed using the scikit-learn package in Python based on the correlation matrix of the standardized variables. Principal components were retained based on cumulative explained variance, selecting those that collectively accounted for more than 90% of the total variance. Component loadings were analyzed to determine the physicochemical contribution to each principal axis, while score plots (PC1 vs. PC2) were utilized to visualize compound distribution within the reduced dimensional space and correlate their spatial clustering with observed antifungal activity.

#### 3.5.4. Statistical Analysis and Interpretation

The relationship between biological activity (pEC_50_) and the principal components was evaluated by linear correlation analysis. Pearson correlation coefficients and the corresponding coefficients of determination (R^2^) were calculated to identify which dimensions of the physicochemical space are most strongly associated with variation in antifungal activity. Correlation analyses and linear regressions were performed in Python using the SciPy and scikit-learn libraries.

#### 3.5.5. Ligand Preparation

The three-dimensional structures of the ligands were generated using Avogadro software (v1.2.0n). Subsequently, geometric optimization and energy minimization were performed using the MMFF94 force field. The resulting conformations were inspected, visualized, and exported for further analyses using BIOVIA Discovery Studio Visualizer (v17.2.0.16349).

#### 3.5.6. Structural Modeling and Active Site Characterization of MfCat2

The three-dimensional model of catalase 2 from *M. fructicola* (MfCat2) was constructed following the methodology previously described [10]. The amino acid sequence of MfCat2 was retrieved from the NCBI database (KAA8570148.1) in FASTA format; this protein, encoded by the EYC84_002478 gene, is annotated as a hypothetical peroxisomal catalase. Structural modeling was performed using the SWISS-MODEL server, employing the catalase from *Botryotinia fuckeliana* (M7TYR4, chain A) as a template, with which a sequence identity of 93.62% and a GMQE value of 0.95 were observed, indicating high reliability of the generated model. The final model, consisting of 522 residues, was exported in PDB format for subsequent analyses.

Identification of the putative active site was carried out using the PrankWeb server [47], which is based on machine learning algorithms. A main pocket (Pocket #1) comprising 22 residues was detected, with a score of 34.37 and an associated probability of 0.938; the geometric center of this pocket was located at coordinates X = 6.73, Y = 3.24, and Z = −10.26 Å. Finally, structural validity and active site localization were corroborated by structural alignment of the MfCat2 model with the crystallized catalase from *Helicobacter pylori* (PDB ID: 1QQW, resolution 2.0 Å), performed in PyMOL v2.5, which confirmed the topological conservation of key catalytic residues in the region corresponding to the identified pocket.

#### 3.5.7. SDH Receptor Preparation

Receptor preparation was performed following, with minor modifications, the methodology previously reported [11]. The three-dimensional structure of succinate dehydrogenase (SDH) was obtained from the Protein Data Bank (PDB ID: 2FBW), corresponding to a crystallographic structure with a resolution of 2.06 Å. Although this enzyme originates from *Gallus gallus*, it was selected due to the high degree of conservation of the residues forming the ubiquinone-binding site among eukaryotic organisms, including phytopathogenic fungi [48,49]. Since no experimental structures of fungal SDH are currently available, avian- and mammalian-derived models have been widely used in studies aimed at elucidating the molecular basis of succinate dehydrogenase inhibitor (SDHI) activity. These models have been shown to be suitable for reproducing relevant ligand-enzyme interactions and are considered appropriate for exploratory molecular docking analyses focused on mechanistic interpretation rather than definitive structural validation [50,51].

#### 3.5.8. Molecular Docking

Molecular docking simulations were carried out using AutoDock 4.2, implementing the Lamarckian Genetic Algorithm as the conformational search method. During the calculations, protein macromolecules were treated as rigid, whereas ligands were considered fully flexible. For each ligand-receptor complex, 50 independent runs were performed, with a limit of 2.5 × 10^7^ energy evaluations per simulation. The resulting conformations were clustered according to their structural similarity using an RMSD threshold of less than 1.0 Å, and the most representative binding pose was selected from the most populated cluster, considering the most favorable predicted binding free energy (ΔG).

For SDH, the search grid was defined around the catalytic site, centered at coordinates X = 14.37 Å, Y = 15.86 Å, and Z = 8.46 Å, with a grid size of 20 × 20 × 20 points. Protocol reliability was verified by redocking of the co-crystallized ligand (CBE), yielding a mean RMSD value of 1.49 Å. Protein-ligand interaction analysis and visualization were performed using Discovery Studio Visualizer.

#### 3.5.9. Molecular Dynamics Simulation Protocol

The best poses obtained from the molecular docking experiments were selected based on the most negative protein-ligand binding energy values and the lowest RMSD values [52,53]. These conformations were subjected to 100 ns molecular dynamics (MD) simulations using NAMD v3.0.1 [53,54,55,56]. Both proteins and ligands were prepared by adding hydrogens at pH 7.4 (physiological pH) using UCSF ChimeraX v1.11 [57,58,59].

The CHARMM36 force field [60,61,62,63] was used for both proteins and ligands; ligand parameters were obtained using the SwissParam algorithm [64]. Simulations were performed in an explicit aqueous environment using the TIP3P water model [65,66], in a 10 × 10 × 10 Å^3^ water box, and the system was neutralized with Na^+^ and Cl^−^ ions at a concentration of 0.15 M. All complexes were subjected to 5000 steps of energy minimization using the conjugate gradient algorithm at 300 K, employing a weak coupling scheme.

Van der Waals interactions were defined with a cutoff radius of 12 Å under the NPT ensemble. Subsequently, the complexes underwent 100,000 equilibration steps, followed by 100 ns of production with an integration time step of 1.0 fs, using the velocity Verlet algorithm. Simulation data were extracted and analyzed using the MDAnalysis, Matplotlib (v3.8.0), and MDTraj Python libraries (Python v3.12).

## 4. Conclusions

In summary, a comprehensive 33-compound library of 3-acyl-bromoindole regioisomers (series **4a**–**k**, **5a**–**k**, and **6a**–**k**) was successfully assembled—including seven novel 4-bromoindole derivatives reported here for the first time (**4d**–**f**, **4h**–**k**)—establishing that bromine regiochemistry dictates antifungal potency, pathogen selectivity, and life-stage targeting. While 3-butyryl-4-bromoindole (**4c**) proved to be the most outstanding broad-spectrum candidate in vitro, postharvest in vivo bioassays on sweet cherries demonstrated that selected bromoindoles (**4**, **6a**, and **6d**) significantly suppressed *M. fructicola* rot severity by 20% to 27% relative to the untreated control. Furthermore, QSAR/PCA, molecular docking, and 100 ns molecular dynamics simulations validated a stable, multi-target mode of action involving both SDH and *M. fructicola* MfCat2. Overall, these structure–activity relationships establish 3-acyl-bromoindoles as highly promising lead scaffolds for the development of modern, multi-site postharvest agrochemicals.

In summary, a comprehensive 33-compound library of 3-acyl-bromoindole regioisomers (series **4a**–**k**, **5a**–**k**, and **6a**–**k**) was successfully assembled—including seven novel 4-bromoindole derivatives reported here for the first time (**4d**–**f**, **4h**–**k**)—establishing that bromine regiochemistry strongly influences antifungal potency, pathogen selectivity, and life-stage response. While 3-butyryl-4-bromoindole (**4c**) exhibited a dual-action profile in vitro, postharvest in vivo bioassays on sweet cherries demonstrated that selected bromoindoles (**4**, **6a**, and **6d**) moderately reduced *M. fructicola* rot severity by 20% to 27% relative to the untreated control. Furthermore, QSAR/PCA, molecular docking, and 100 ns molecular dynamics simulations provided preliminary evidence supporting plausible interactions with both SDH and *M. fructicola* MfCat2. Overall, these structure–activity relationships establish 3-acyl-bromoindoles as exploratory chemical leads that require further optimization, formulation, and toxicological validation for potential postharvest application.

## Figures and Tables

**Table 1:** In vitro antifungal activity of 4-bromoindole (**4**) and 3-acyl-4-bromoindole derivatives (**4a**–**k**).

Compound	*B. cinerea*		*M. fructicola*	
	EC_50_ (µg/mL)	ICG (%)	EC_50_ (µg/mL)	ICG (%)
**4**	>250	NI	7.05 ± 1.61 ^b^	0
**4a**	>250	NI	>250	NI
**4b**	>250	NI	>250	NI
**4c**	127.27 ± 0.74 ^c^	98	25.00 ± 0.32 ^e^	86
**4d**	>250	NI	30.81 ± 0.63 ^f^	22
**4e**	186.53 ± 0.81 ^e^	75	>250	NI
**4f**	168.88 ± 0.79 ^d^	52	16.74 ± 0.19 ^d^	19
**4g**	>250	NI	199.31 ± 0.82 ^g^	2
**4h**	>250	NI	>250	NI
**4i**	>250	NI	>250	NI
**4j**	>250	NI	>250	NI
**4k**	201.83 ± 0.83 ^f^	0	12.21 ± 3.30 ^c^	0
BC-1000^®^	95.13 ± 0.67 ^b^	100	5.05 ± 1.5 ^b^	84
Captan^®^	82.89 ± 0.69 ^a^	100	-	-
Mystic^®^ 520 SC	-	-	0.70 ± 0.0 ^a^	85

Values are the mean ± standard deviation (SD) of three replicates. “-” means no experiment is carried out. NI: No inhibitory effect on conidial germination at 250 µg/mL. Statistical significance was evaluated using one-way ANOVA followed by Fisher’s LSD test (*p* < 0.05), except for compounds with EC_50_ values >250 µg/mL. Different letters indicate significant differences among treatments.

**Table 2:** Predicted binding energies (ΔG, kcal/mol) and key ligand–protein interactions of the selected bromoindole derivatives with MfCat2 and SDH.

Target	Compound	Binding Energy	Interactions
**MfCat2**	**4**	−7.14	Gly165 (1.87 Å); Hsd93; Ala151; Val164
	**4k**	−7.82	Gly 165 (1.87 Å); Arg376; Hsd93; Leu372 Val239; Val164; Ala151
	**5**	−8.96	Asn166 (2.07 Å); Gly165 (2.02); Hsd93; Val164
	**6**	−8.78	Gly165 (1.82 Å); Val164; Ala151; Hsd93
**SDH**	**4**	−7.68	His209 (1.97 Å); His41; His104; Arg42; Ser38
	**4k**	−7.77	Tyr56 (1.86); Trp166 (2.73); Ile26; Ile39; Met35; Trp165; Trp31
	**5**	−6.81	Arg42; His209; Asp55; His104
	**6**	−8.96	His355 (2.79 Å); Arg288; Arg399; Ala51

## Data Availability

The original contributions presented in this study are included in the article and Supplementary Materials. Further inquiries can be directed to the corresponding author.

## Supplementary Materials

The following supporting information can be downloaded at https://www.mdpi.com/article/10.3390/antibiotics15080801/s1, File S1: ^1^H NMR spectrum/^13^C NMR spectrum/Mass spectra of compounds. File S2: In vivo assay of compound series **4**–**6**. Table S1: Physicochemical descriptor values used for principal component analysis, along with corresponding PC1 and PC2 scores for *B. cinerea*. Table S2: Physicochemical descriptor values used for principal component analysis, along with PC1 and PC2 scores for *M. fructicola*. Table S3: Experimental reaction parameters (temperature and time) for 4-bromoindole derivatives (**4a**–**k**).
