# Supplementary material for: Synthesis and In Vivo Antifungal Evaluation of 3-Acyl-bromoindole Regioisomers: A Multi-Targeting Study on Postharvest Pathogen Control and Molecular Dynamics
Source: Antibiotics (Basel). 2026 Aug 18;15(8):801. doi: 10.3390/antibiotics15080801 (PMC13509852; doi:10.3390/antibiotics15080801)
Supplement: Supplementary file 1 [file antibiotics-15-00801-s001.zip › antibiotics-4506290-supplementary.pdf]

SUPPORTING INFORMATION

Synthesis and In Vivo Antifungal Evaluation of 3-Acyl-Bromoindole Regioisomers: A Multi-Targeting Study on Postharvest Pathogen Control and Molecular Dynamics

Alejandro Madrid [1,*], Valentina Silva [1], Katy Diaz [2] , Evelyn Muñoz [3], David Cabezas [4]; Karel Mena [5, 6], Iván Montenegro [7], Carmina Sirignano [8], Enrique Werner [9] , and Ximena Besoain [10]

File S1: ¹H NMR spectrum/¹³C NMR spectrum/Mass spectra of compounds

¹H NMR spectrum of compound **4d**

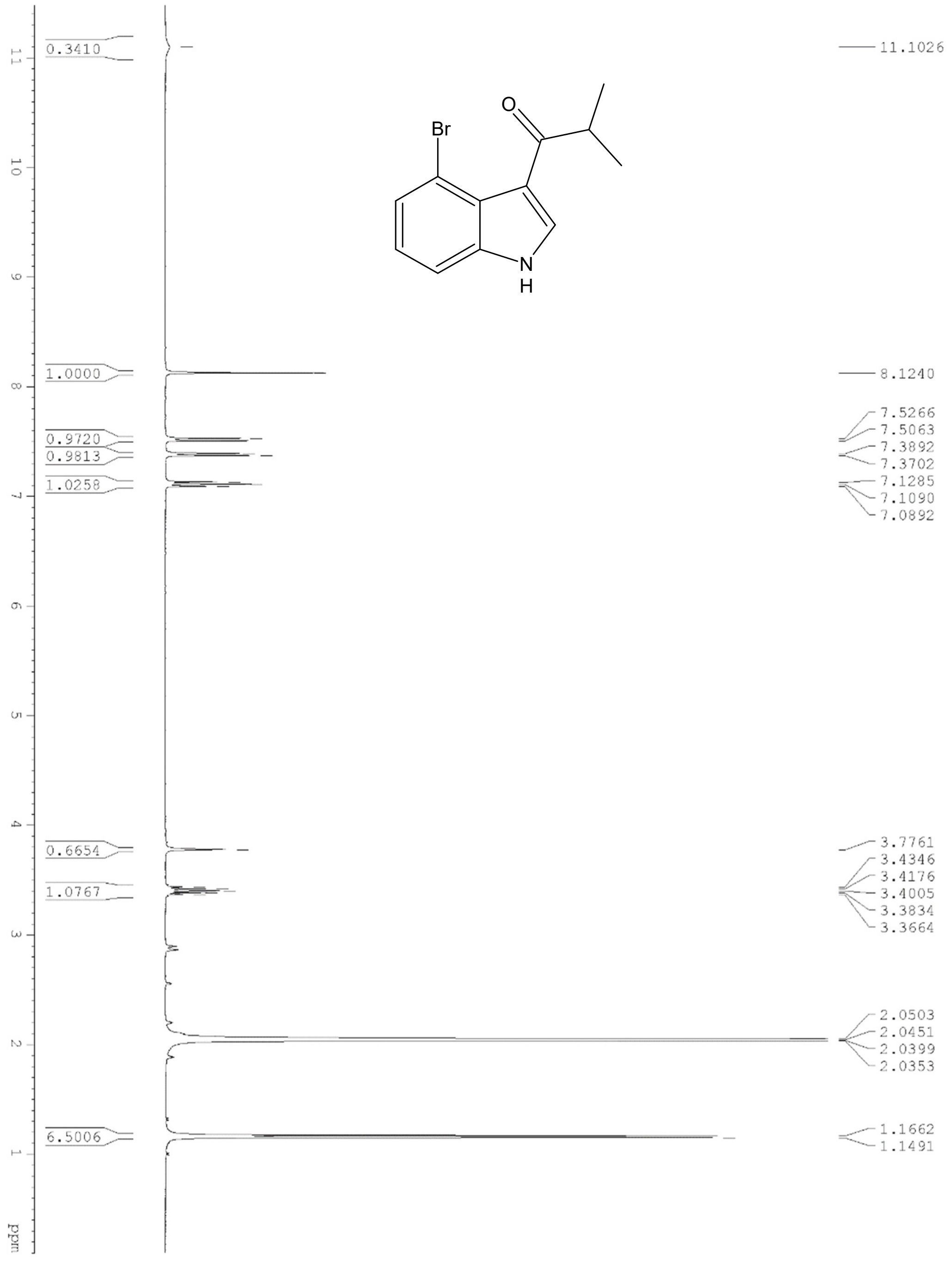

^{13}C NMR spectrum of compound **4d**

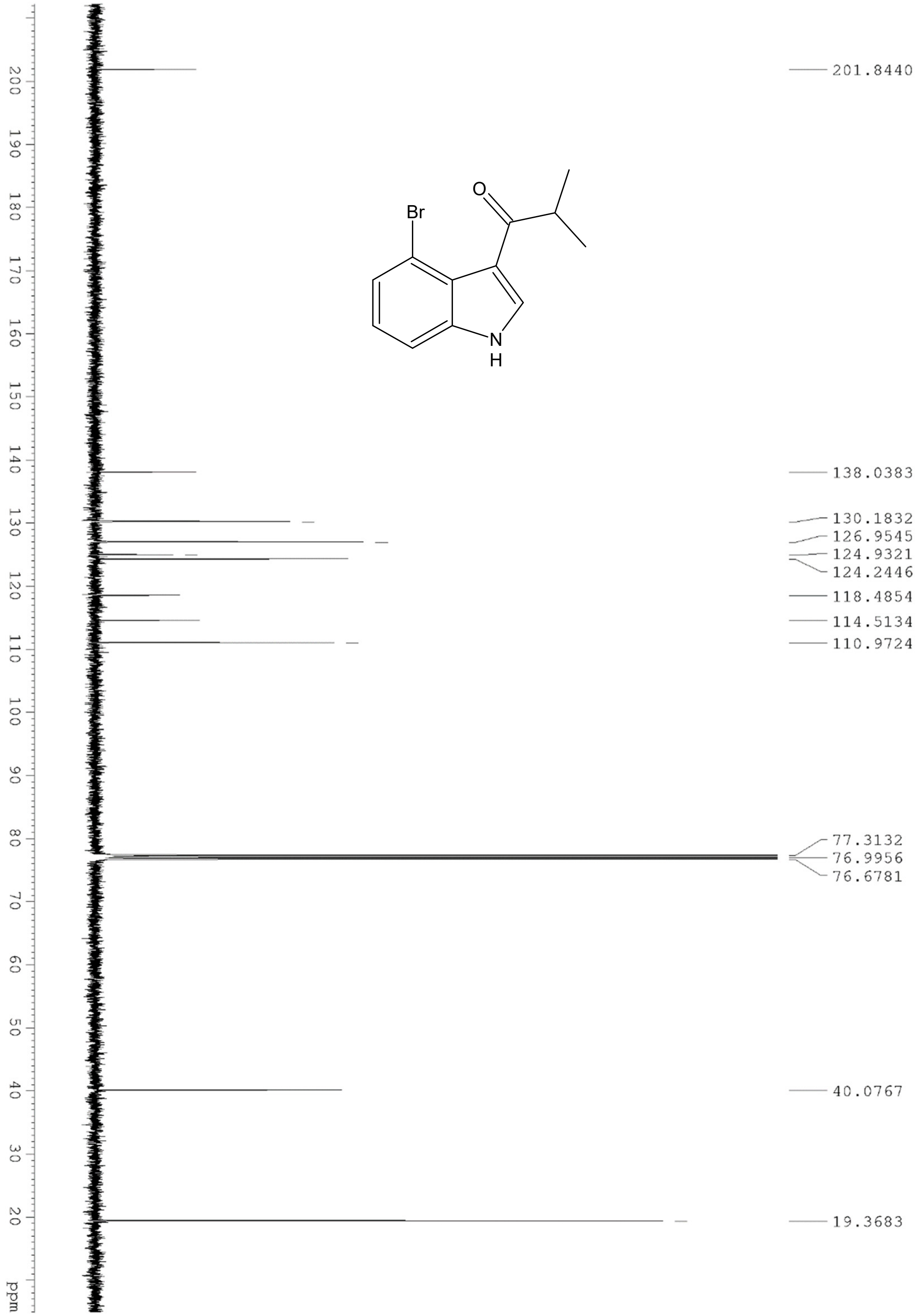

Mass spectra of compound **4d**

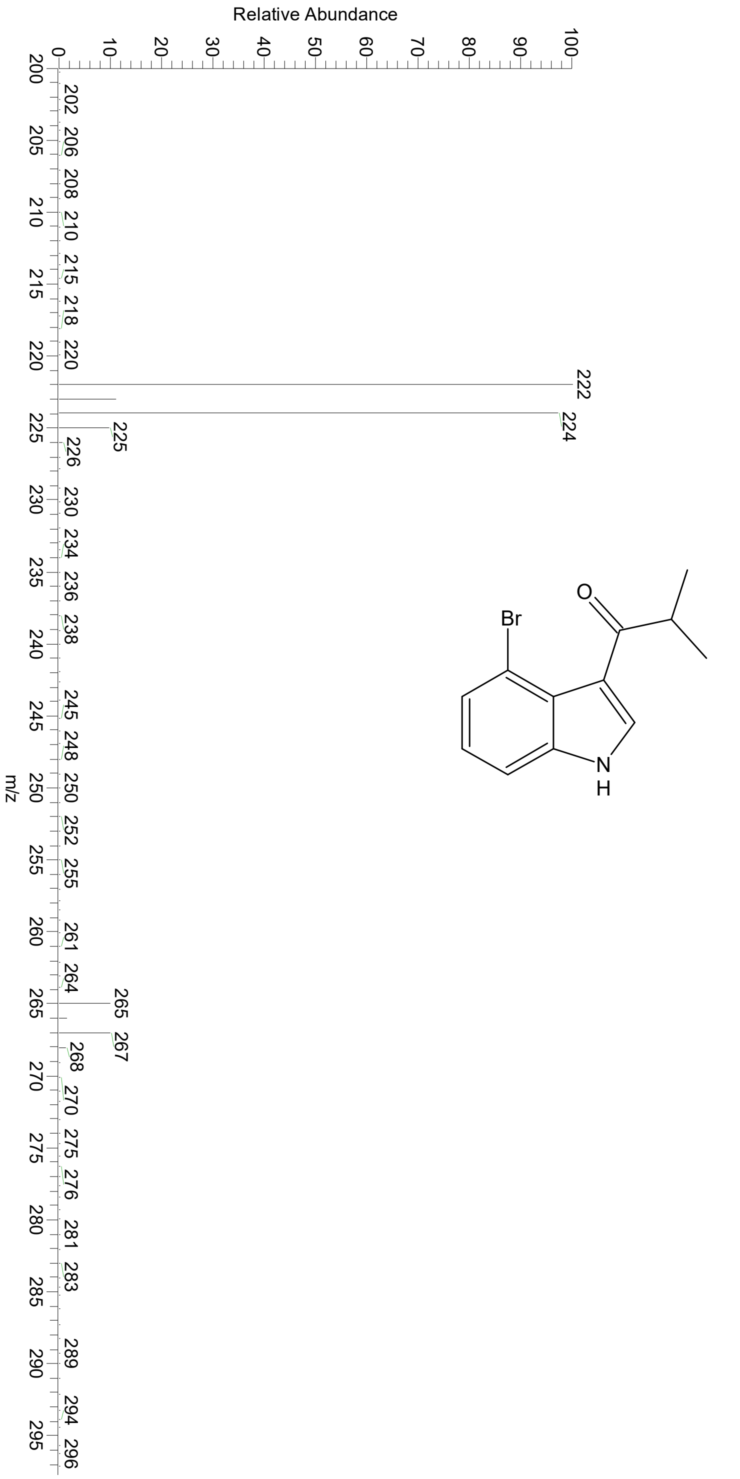

^{1}H NMR spectrum of compound **4e**

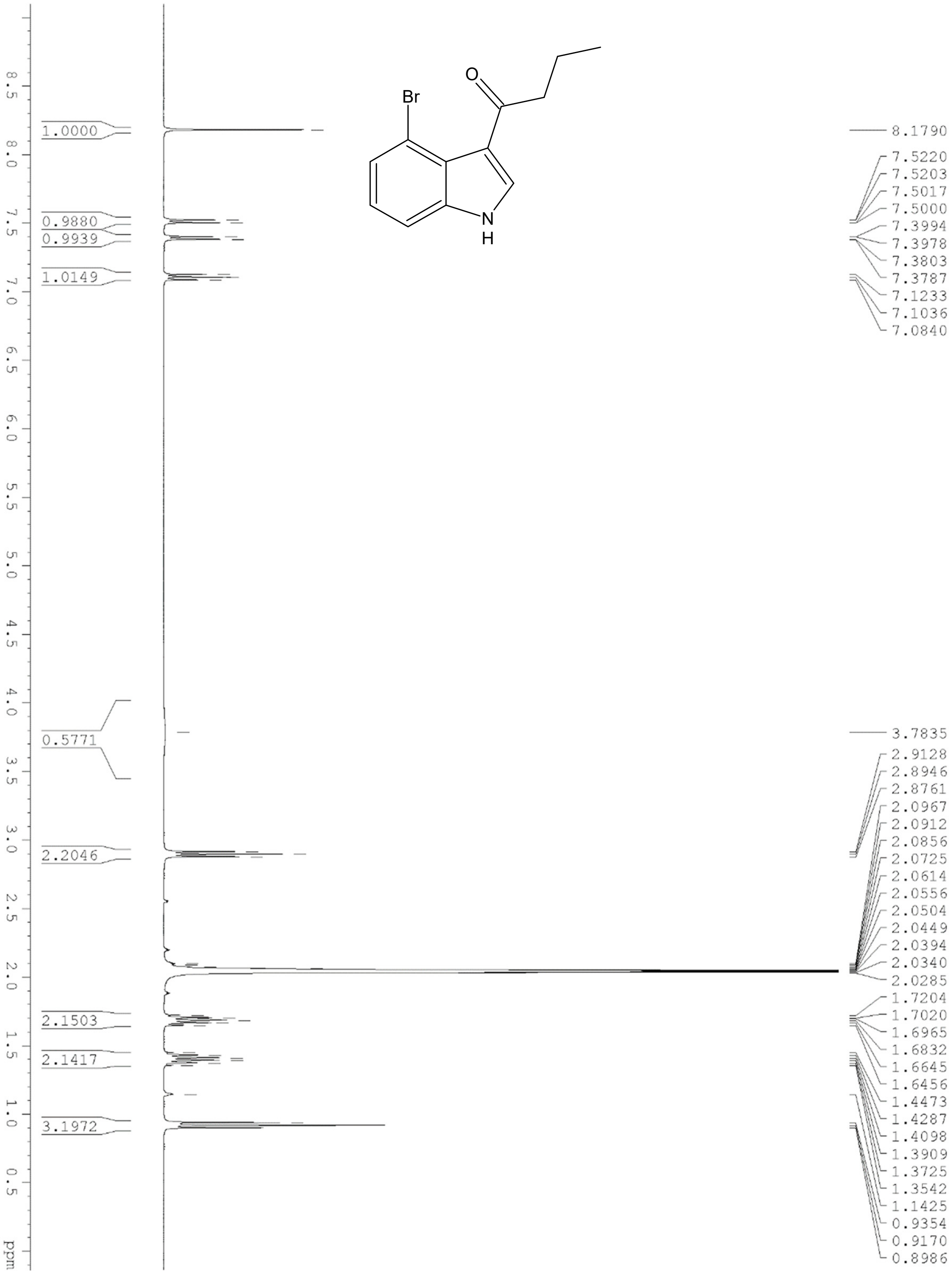

^{13}C NMR spectrum of compound **4e**

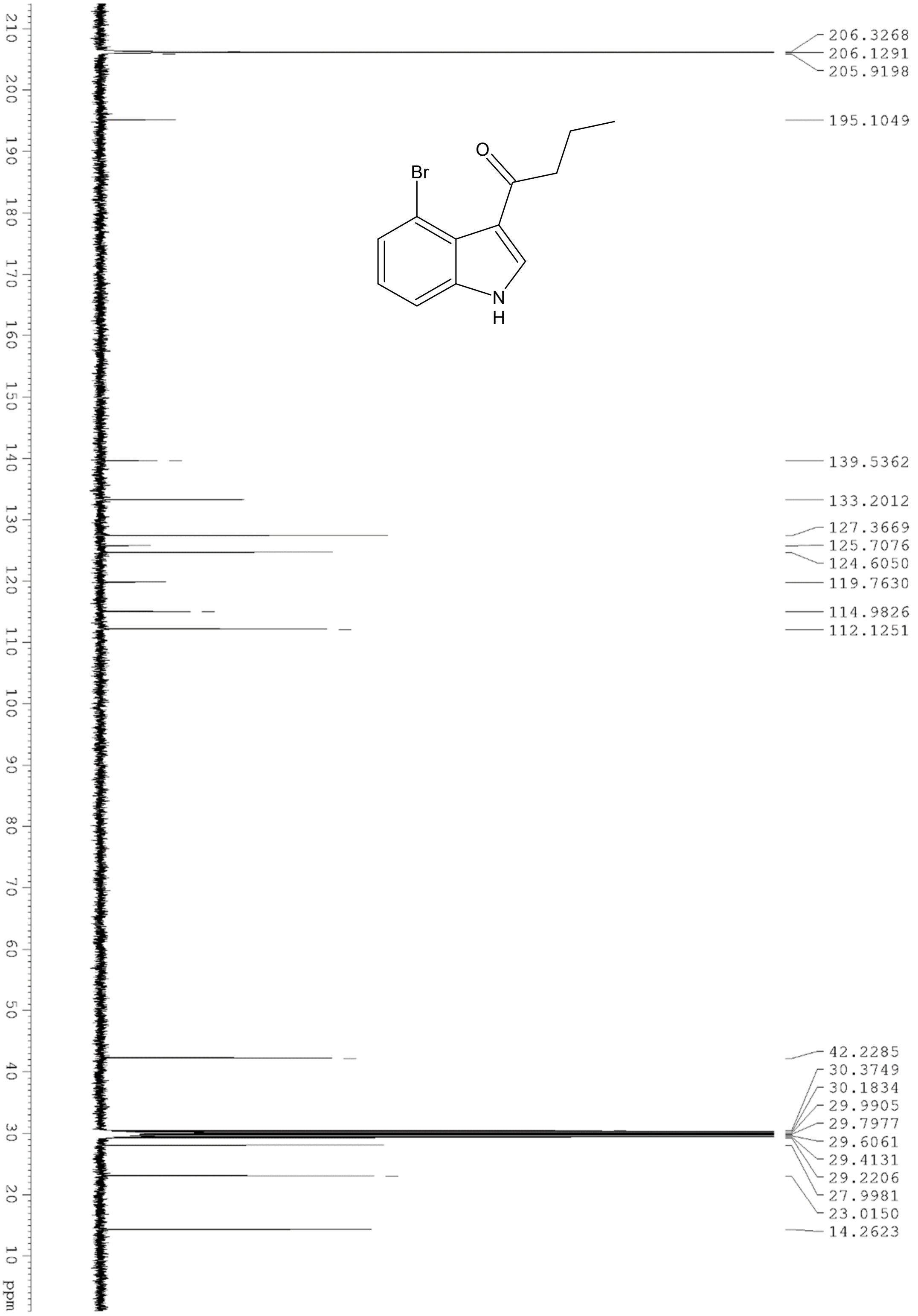

Mass spectra of compound **4e**

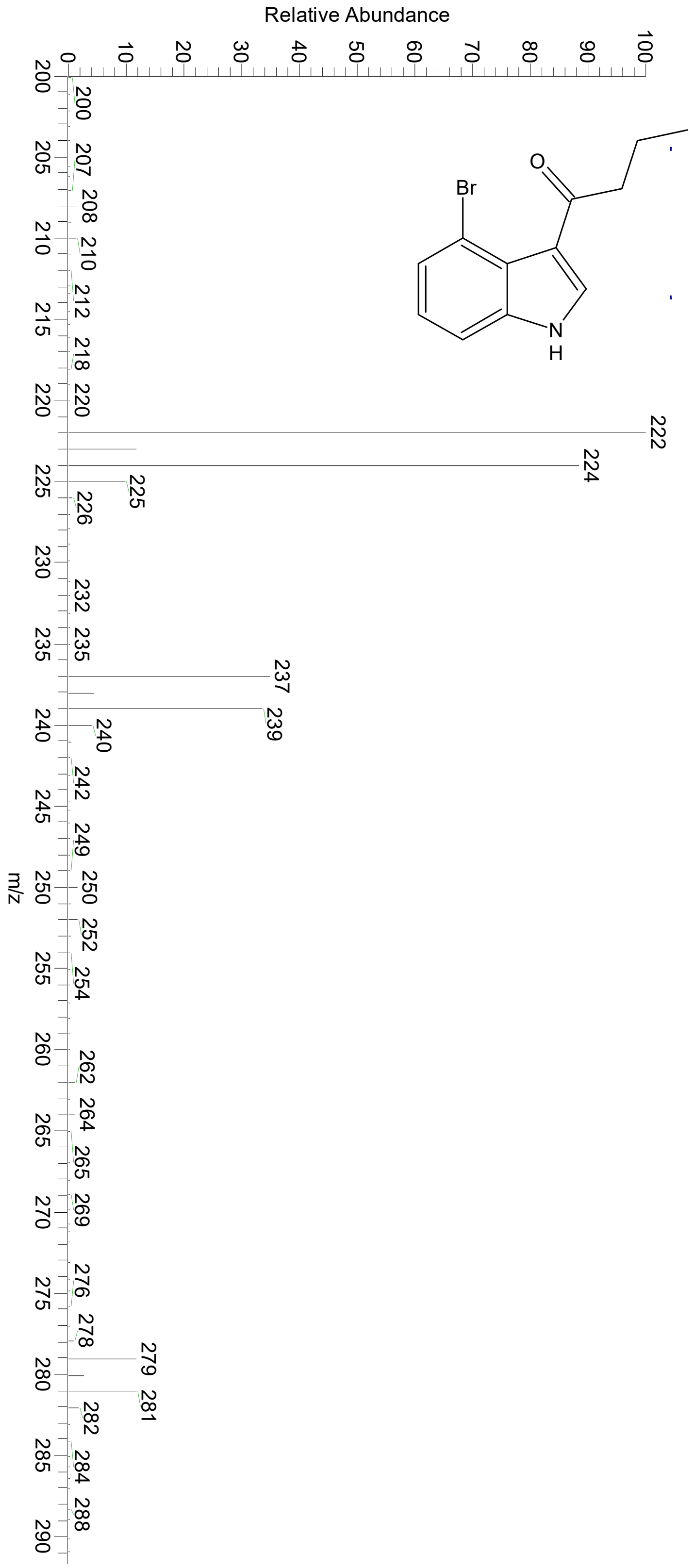

^{1}H NMR spectrum of compound **4f**

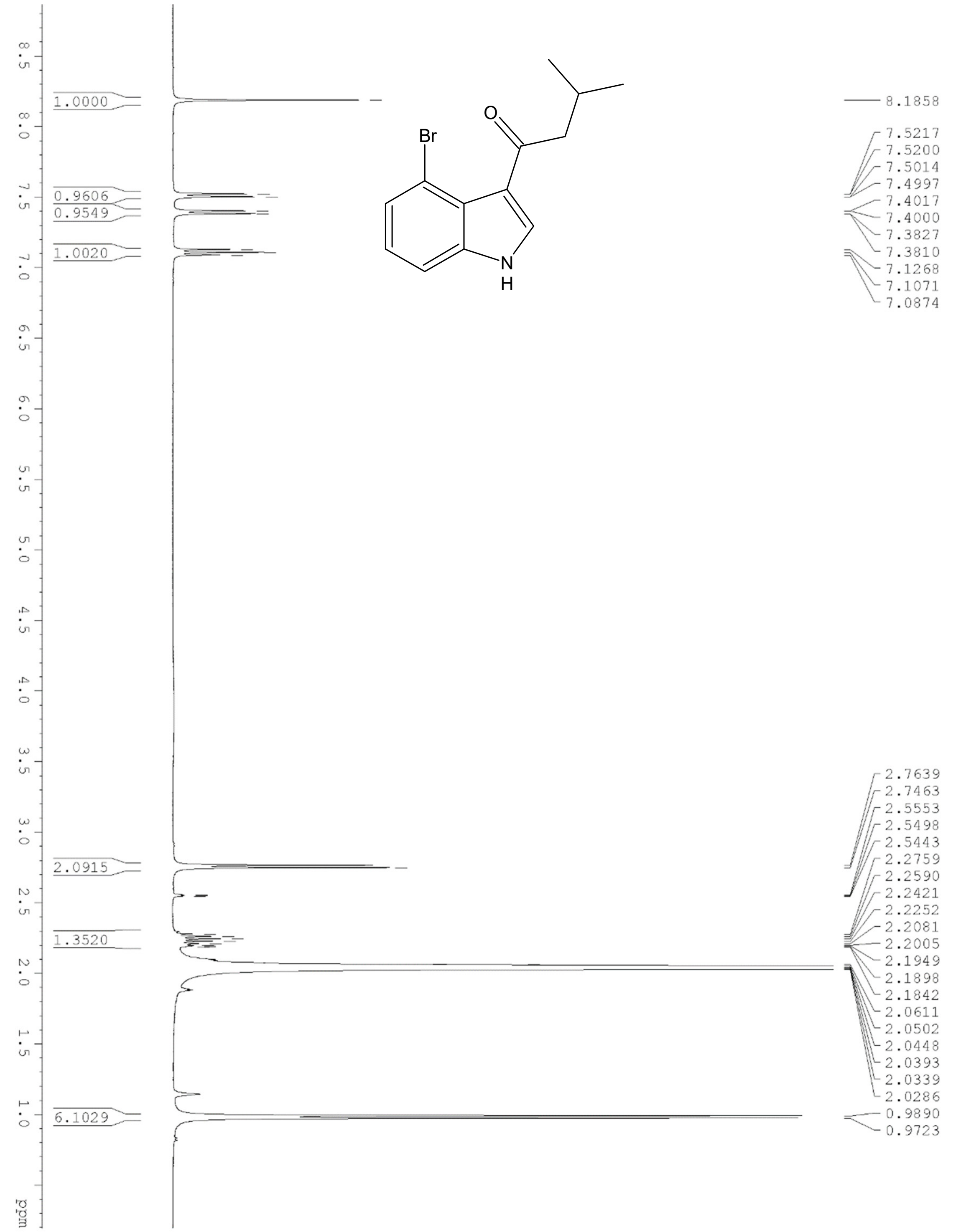

^{13}C NMR spectrum of compound **4f**

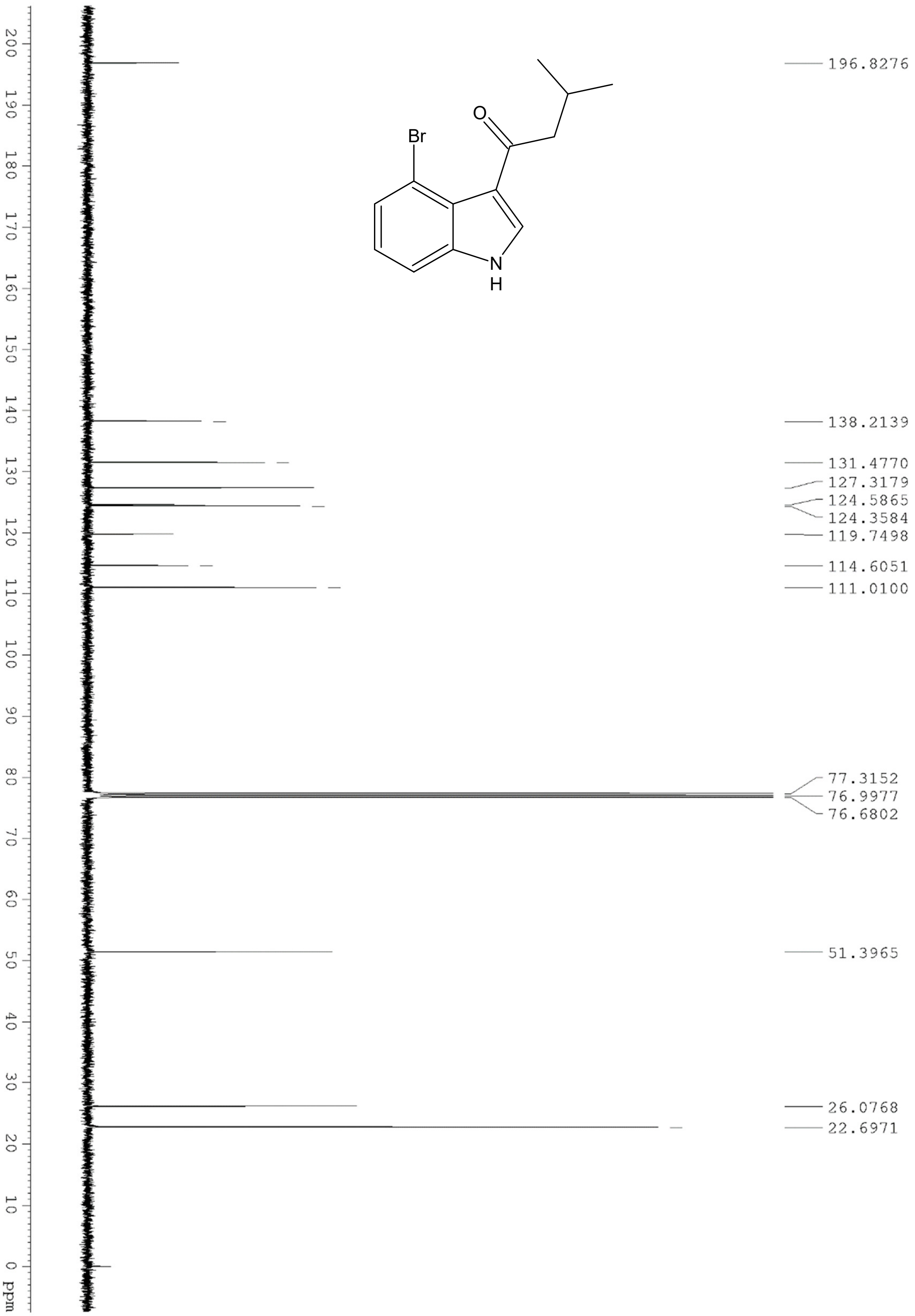

Mass spectra of compound **4f**

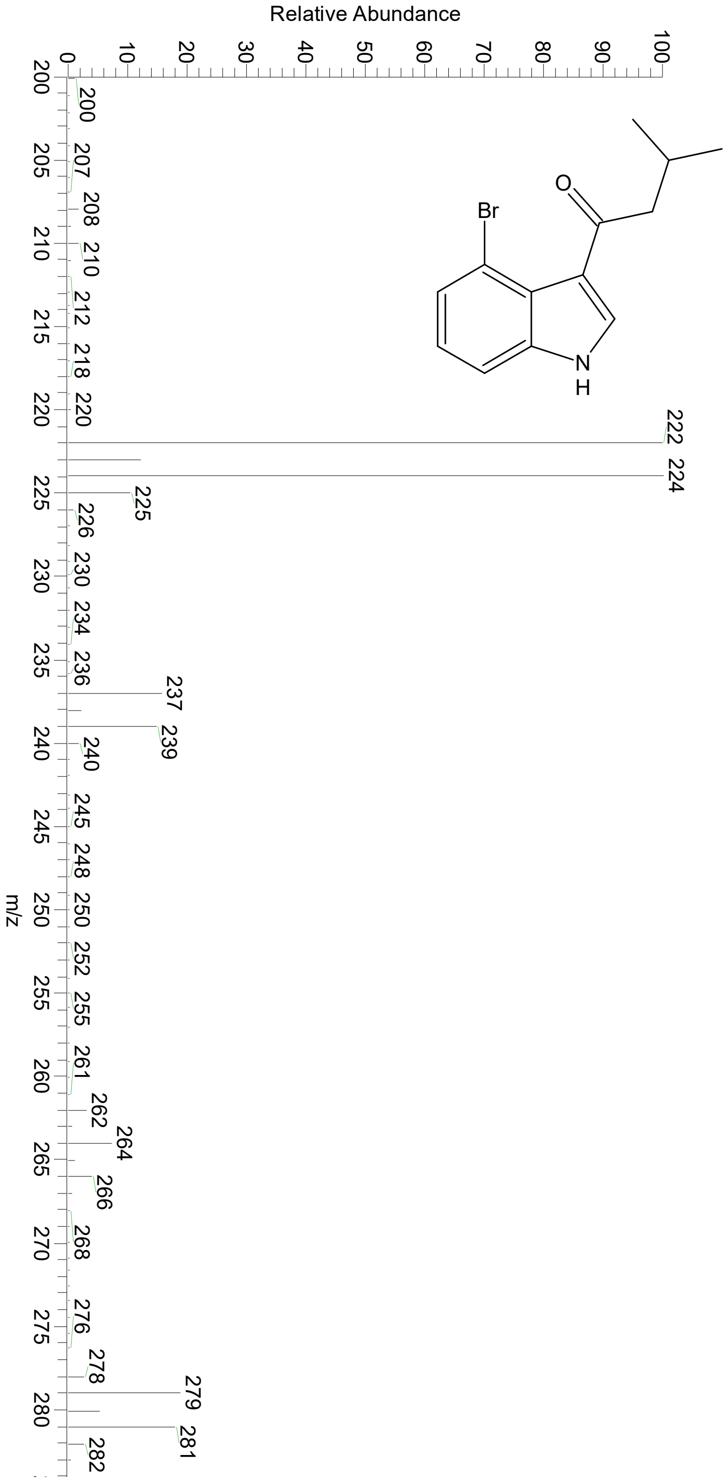

^{1}H NMR spectrum of compound **4h**

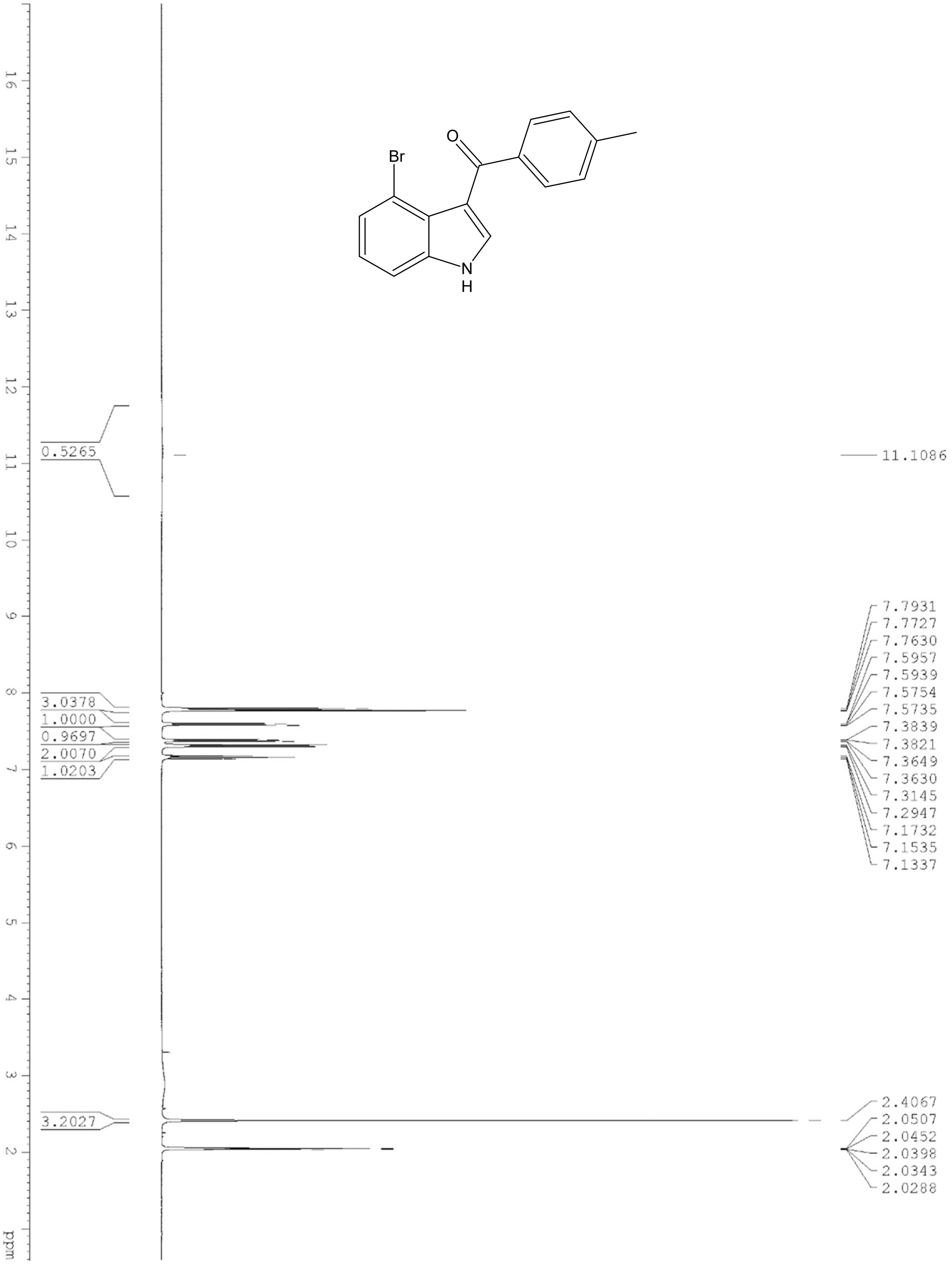

^{13}C NMR spectrum of compound **4h**

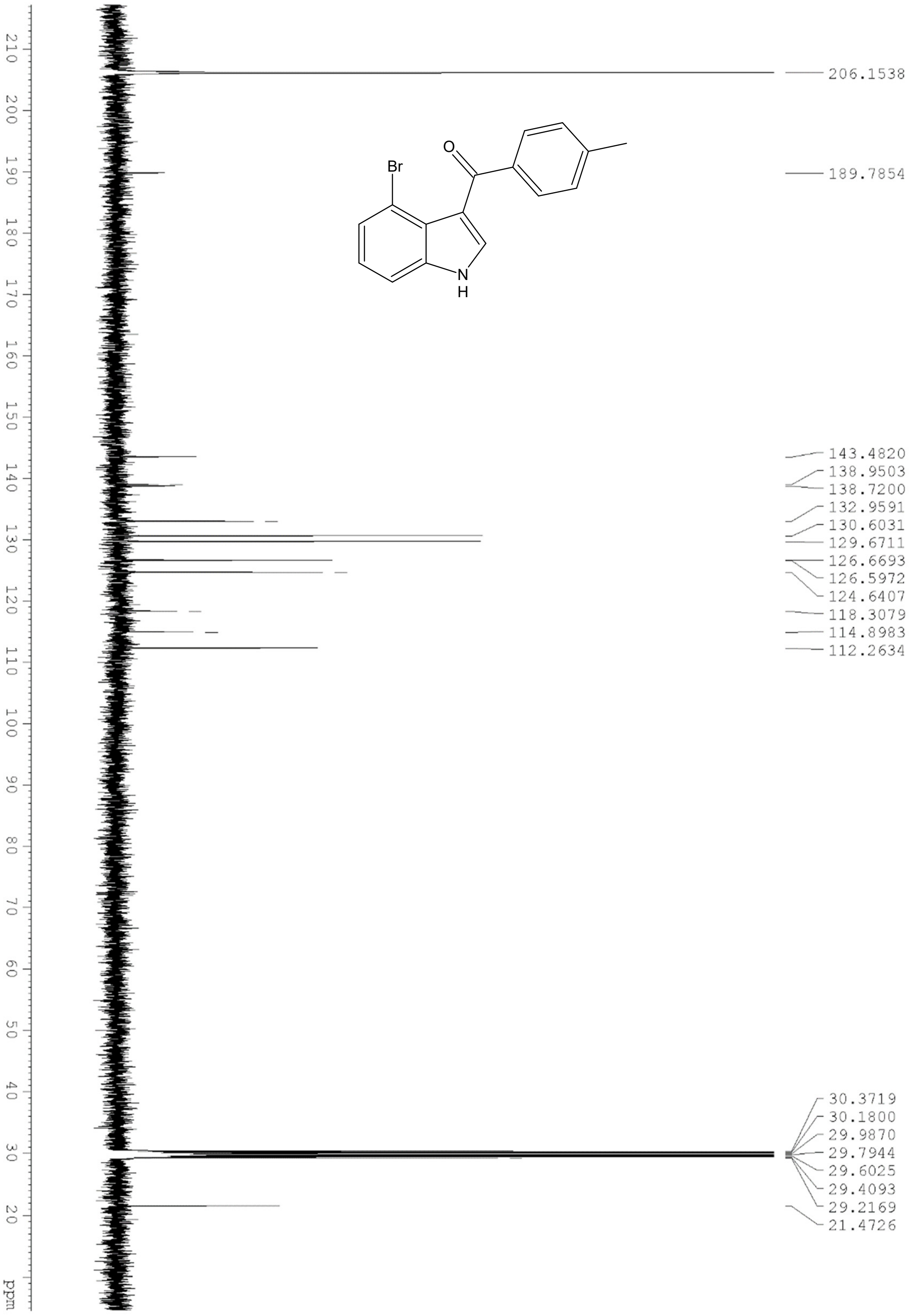

Mass spectra of compound **4h**

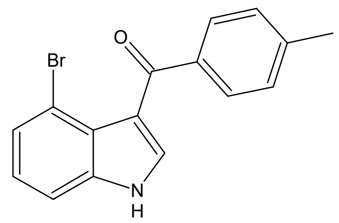

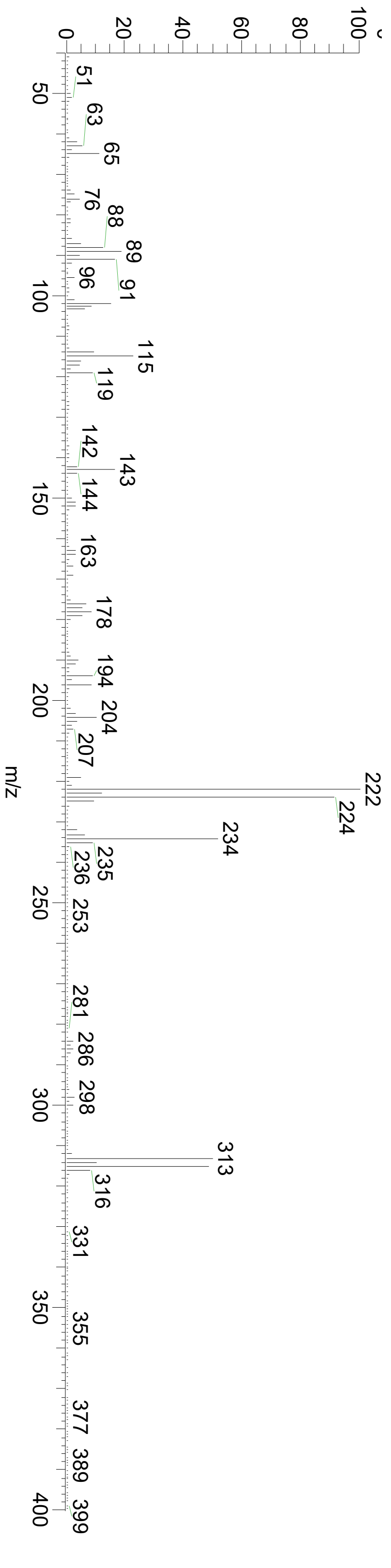

^{1}H NMR spectrum of compound **4i**

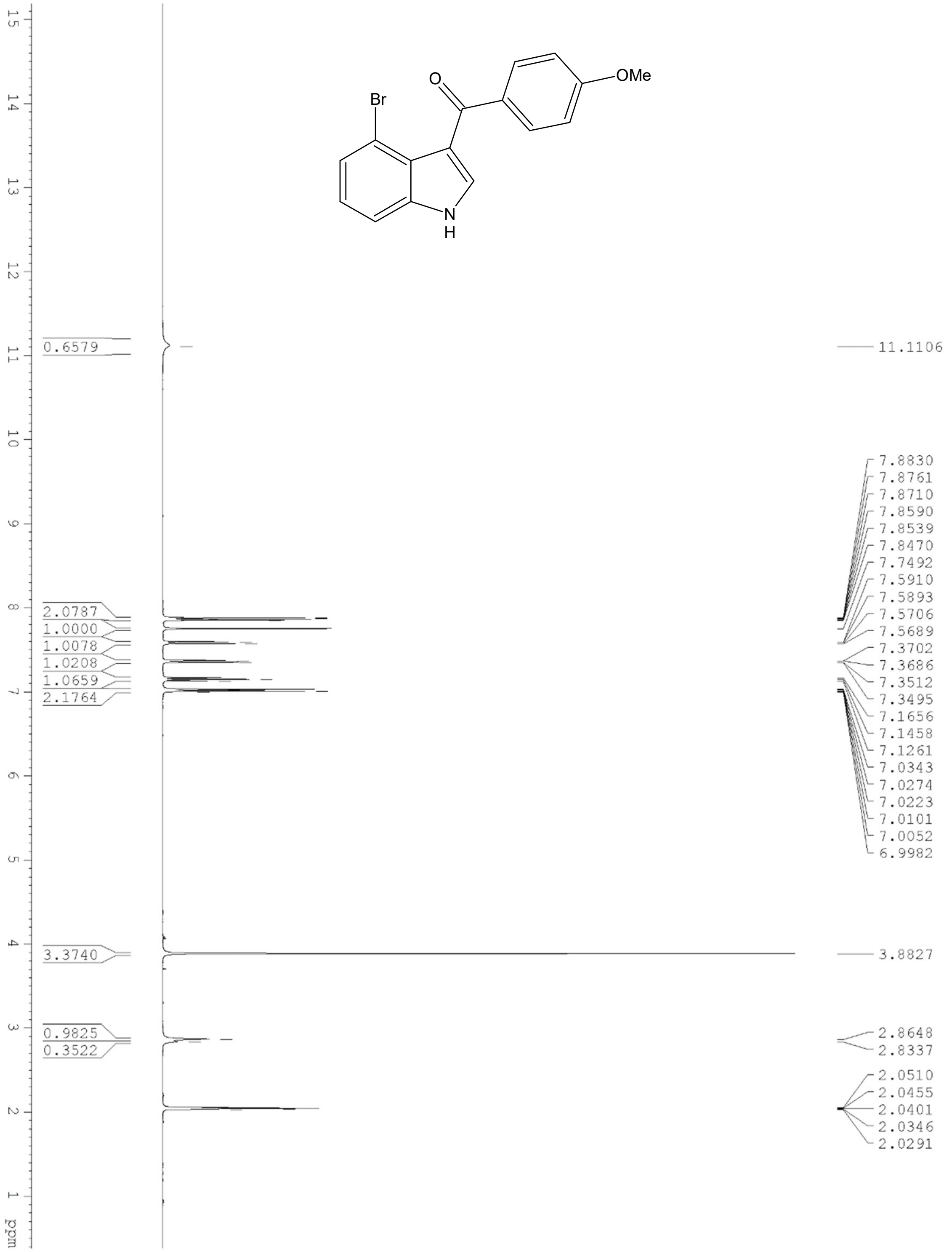

^{13}C NMR spectrum of compound **4i**

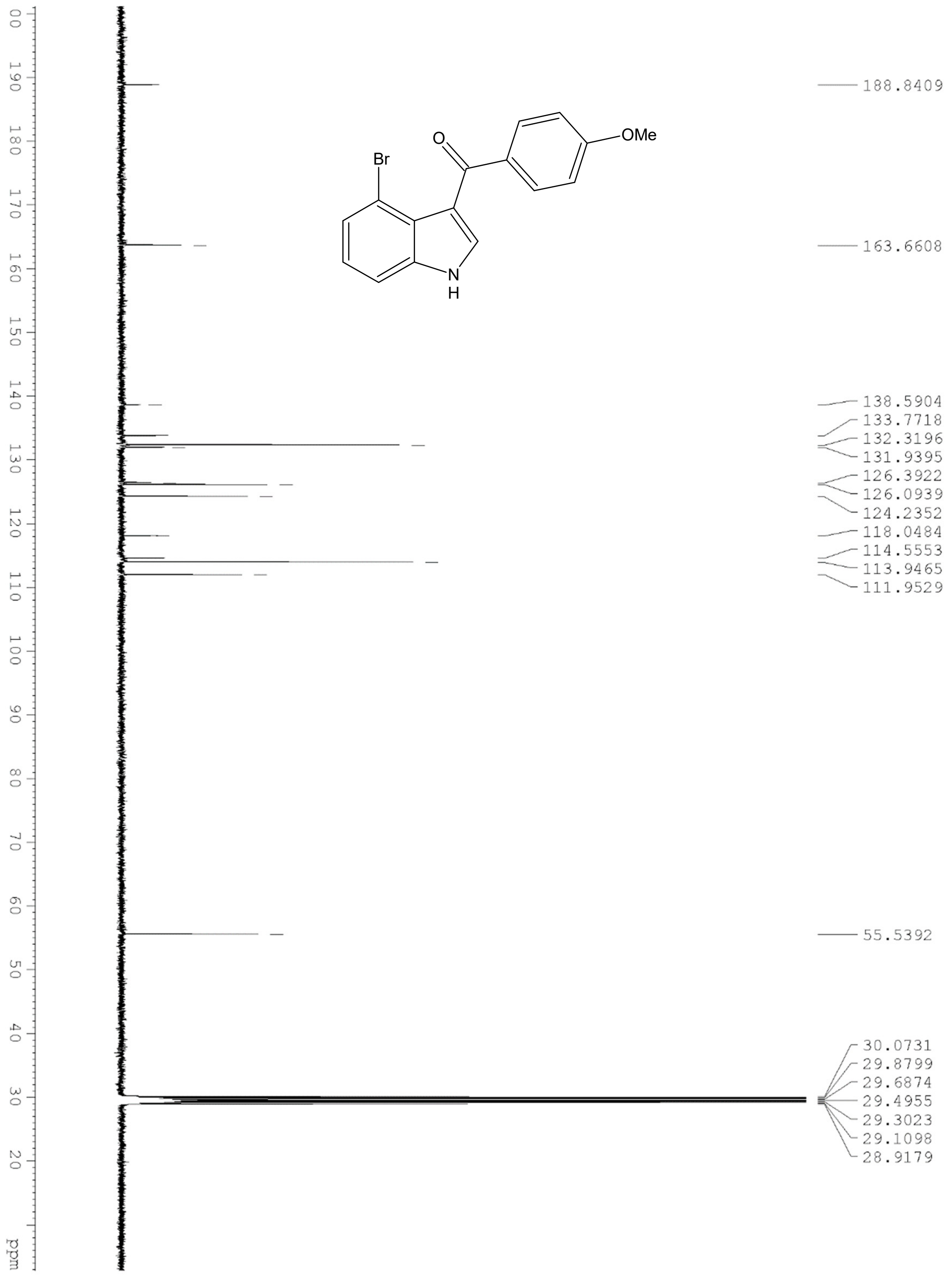

Mass spectra of compound **4i**

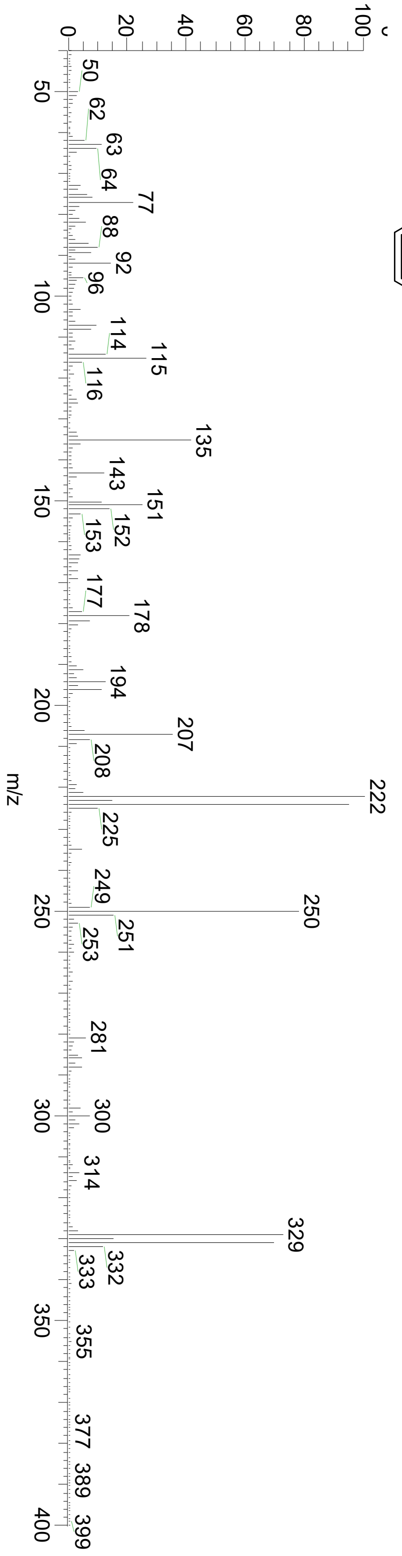

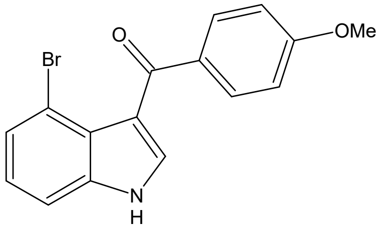

^{1}H NMR spectrum of compound **4j**

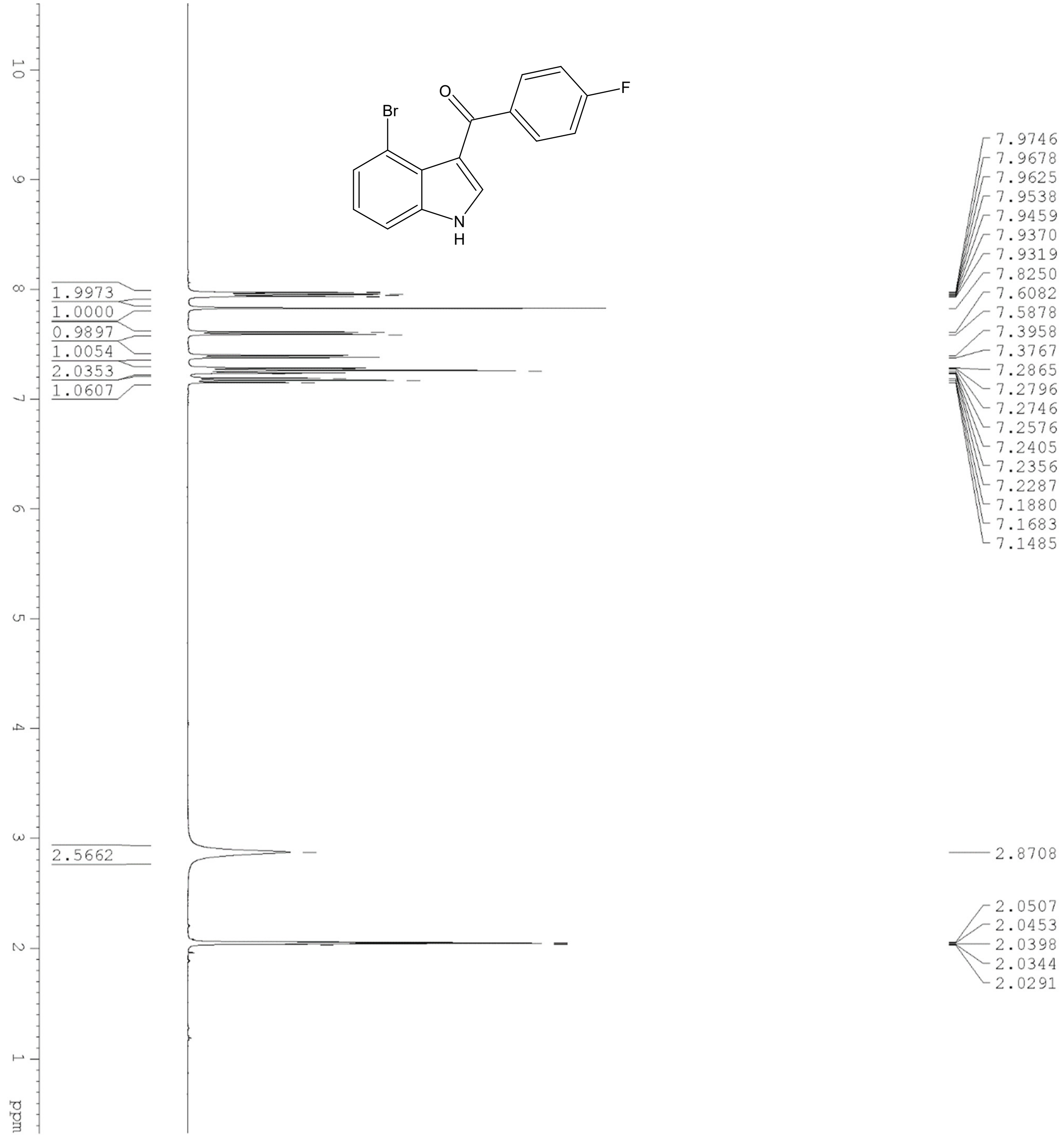

^{13}C NMR spectrum of compound **4j**

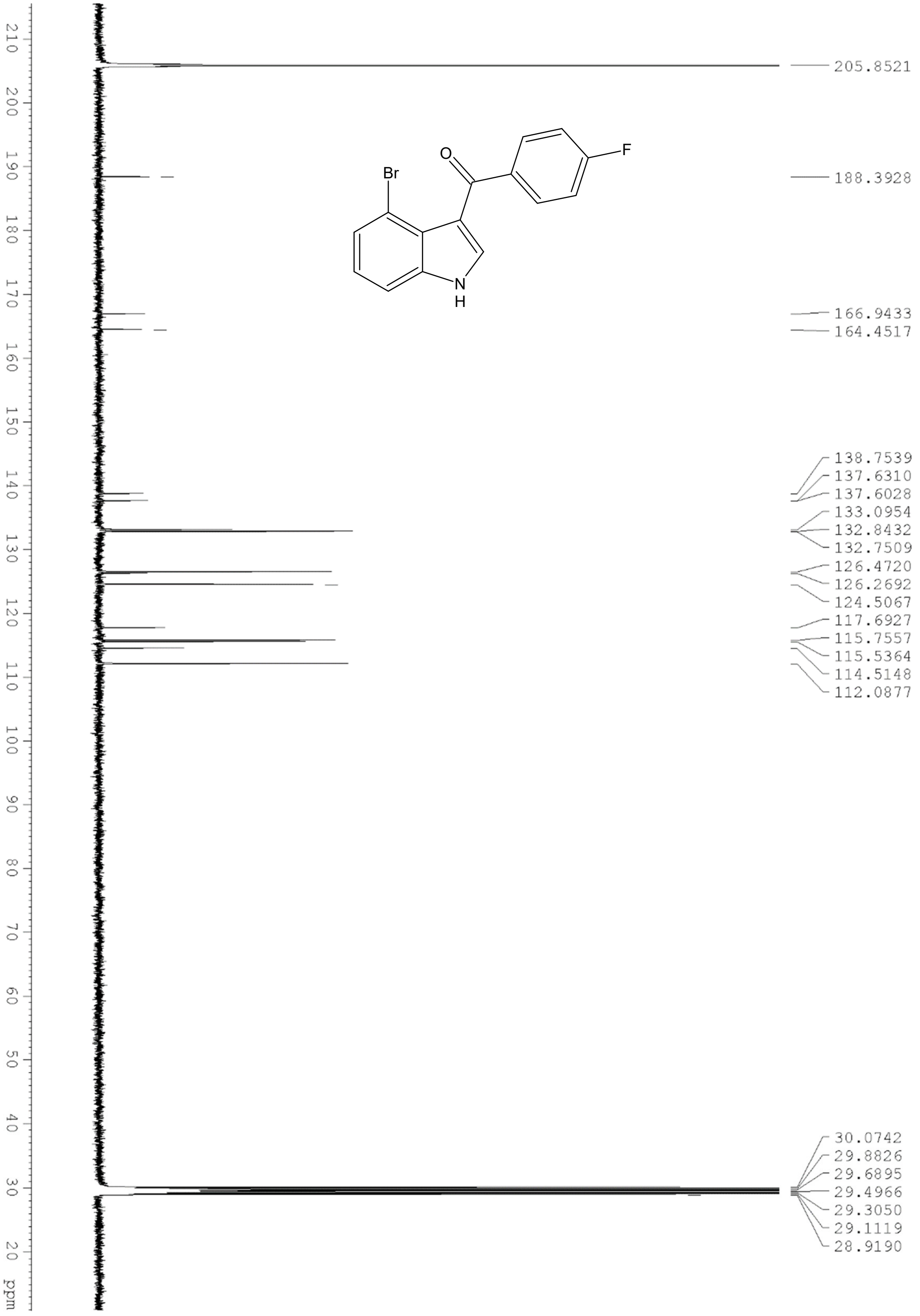

Mass spectra of compound **4j**

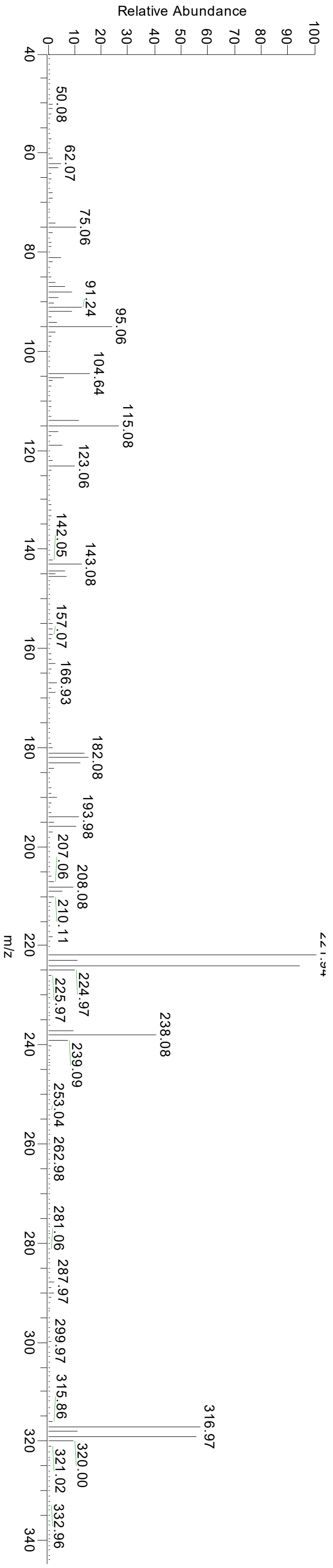

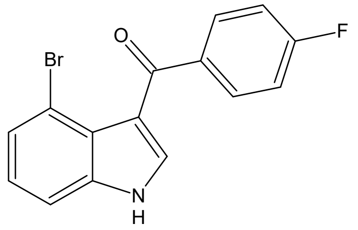

^{1}H NMR spectrum of compound **4k**

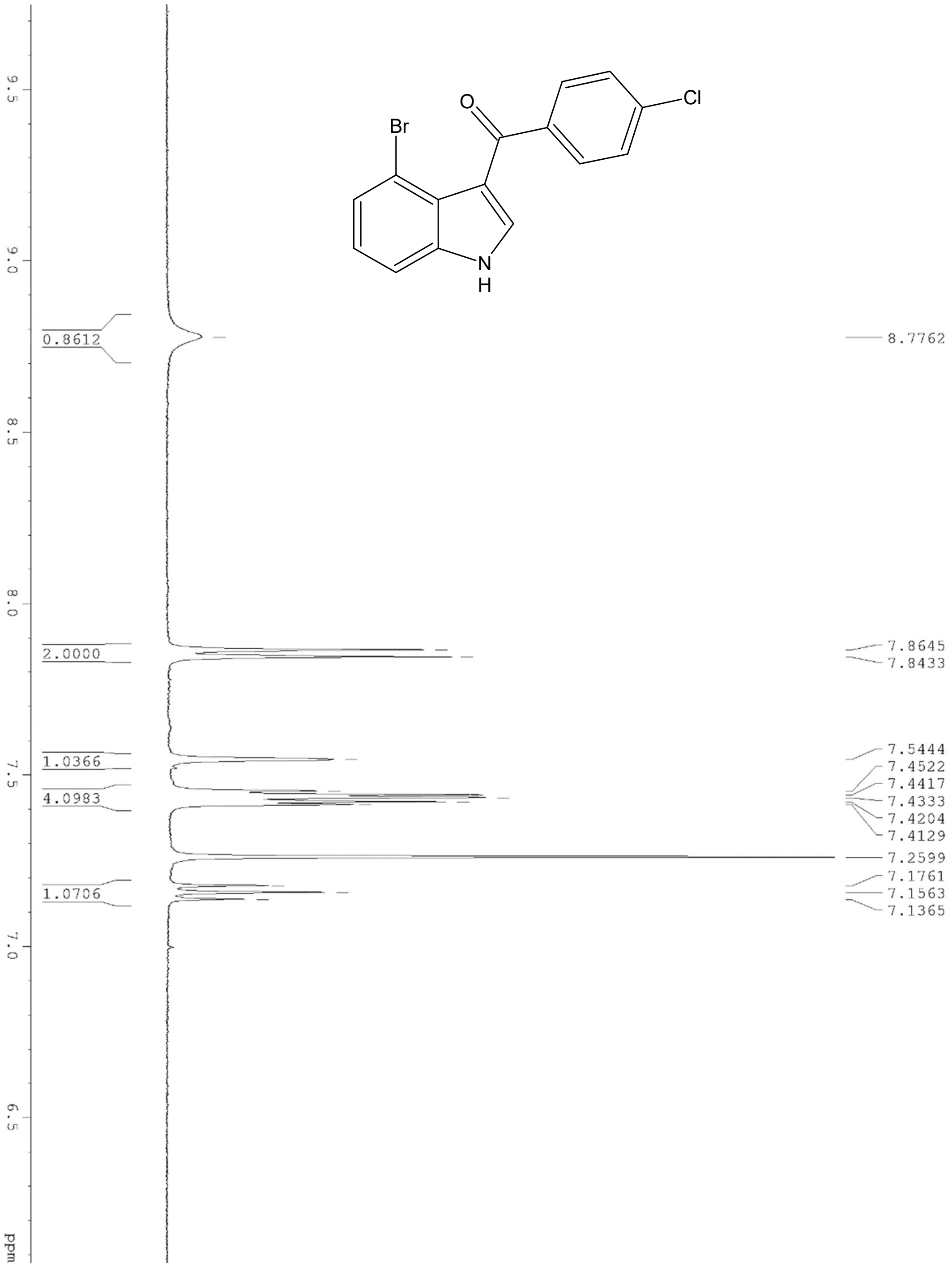

13C NMR spectrum of compound **4k**

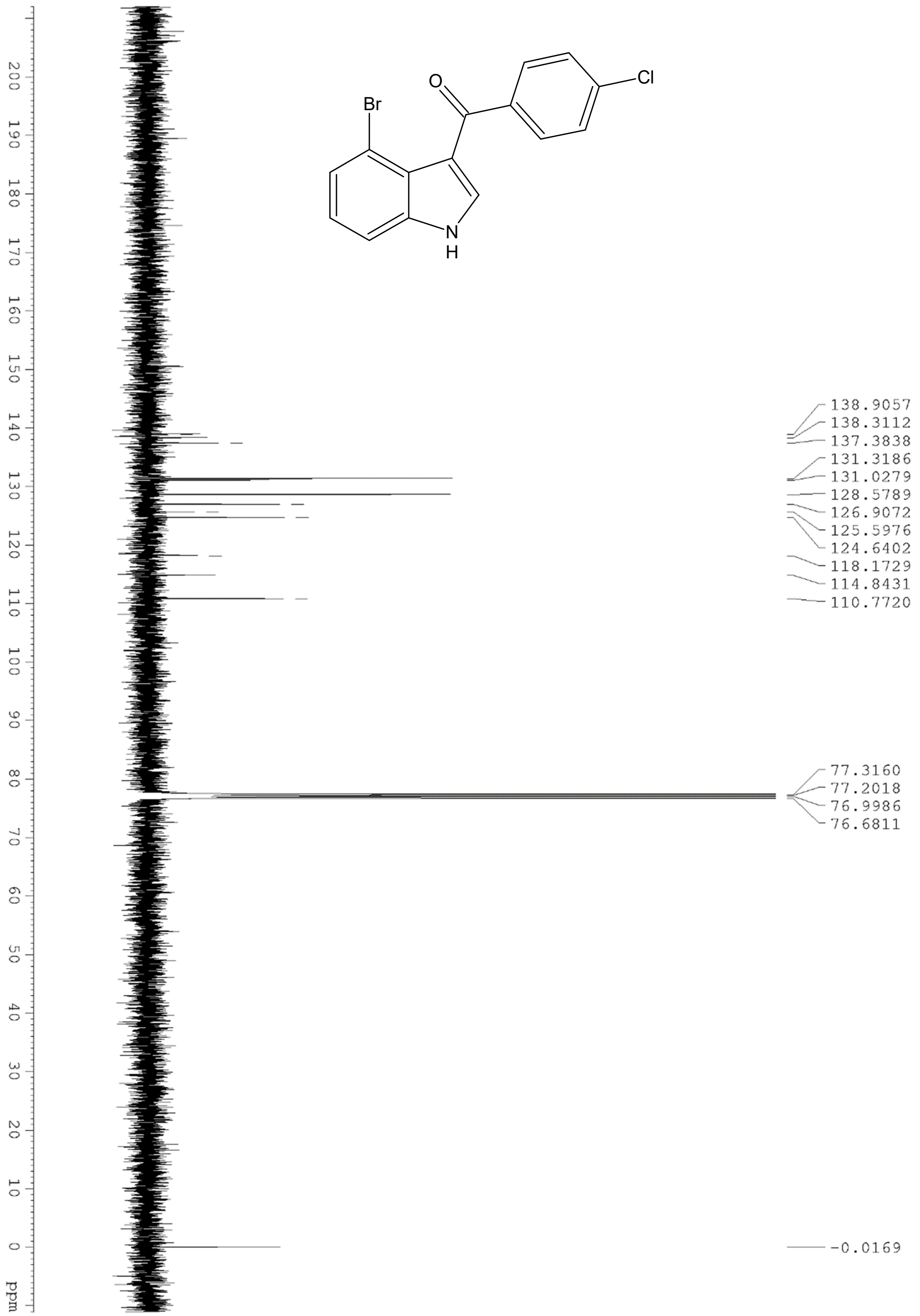

Mass spectra of compound **4k**

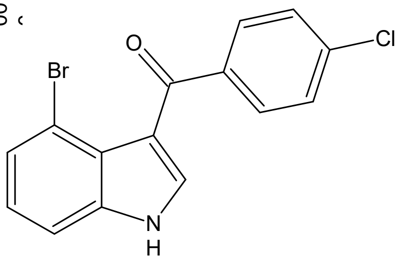

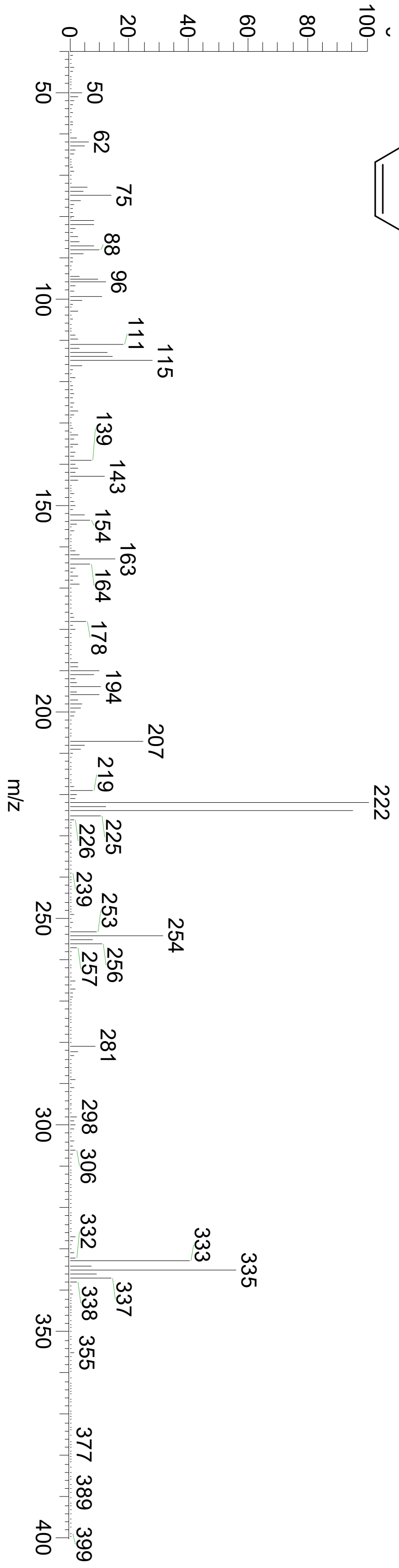

File S2: In vivo assay of compound series 4–6.

in vivo assay of compound series 4

4

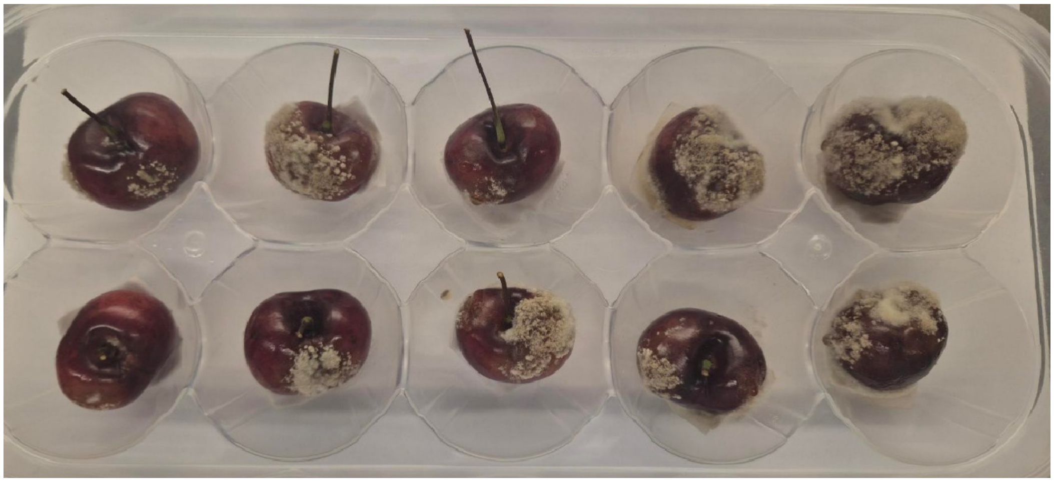

4c

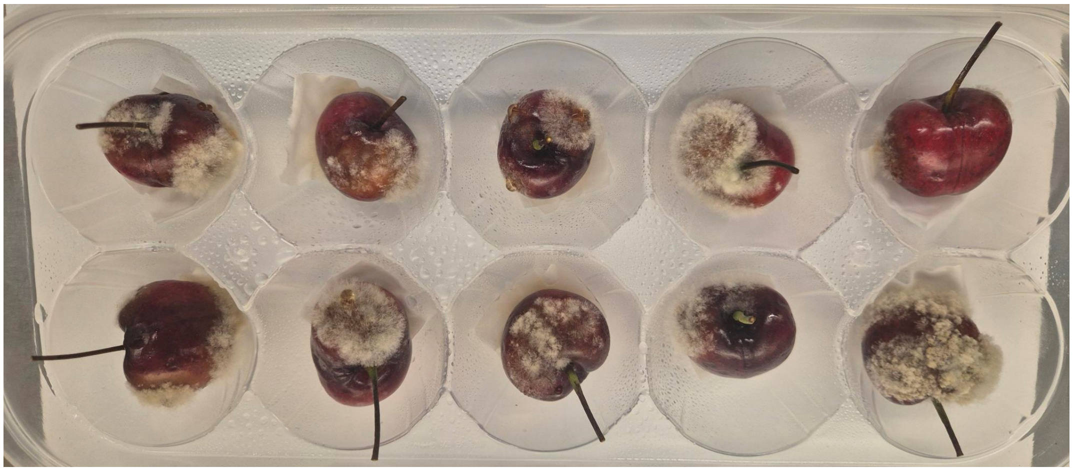

4d

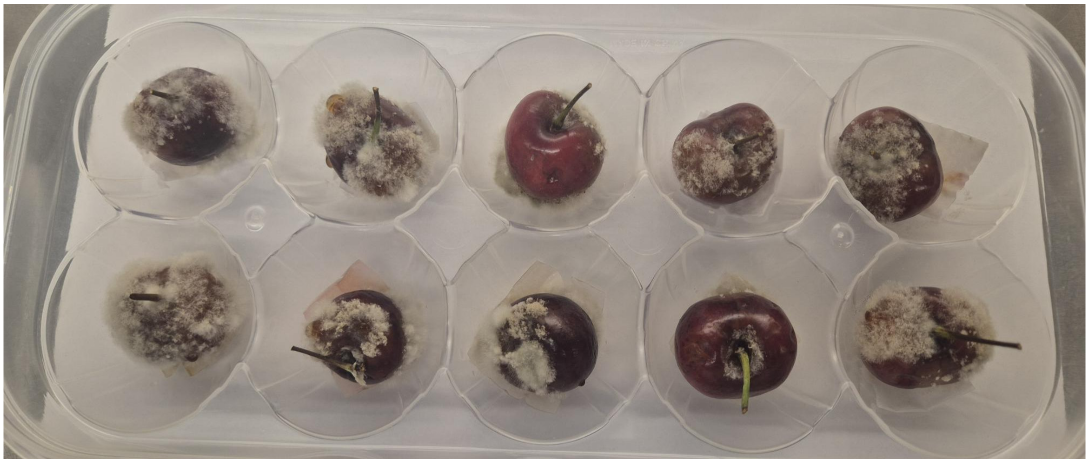

4g

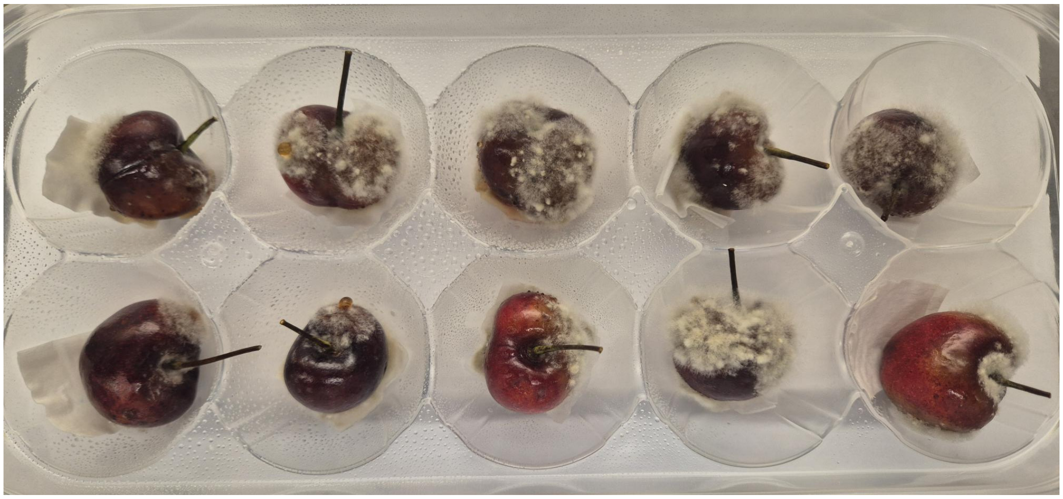

4k

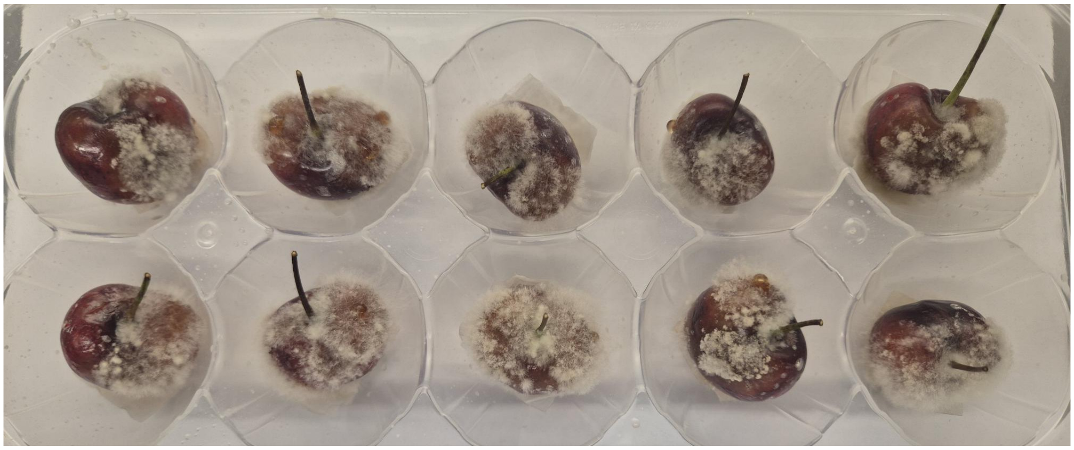

***in vivo* assay of compound series 5**

5

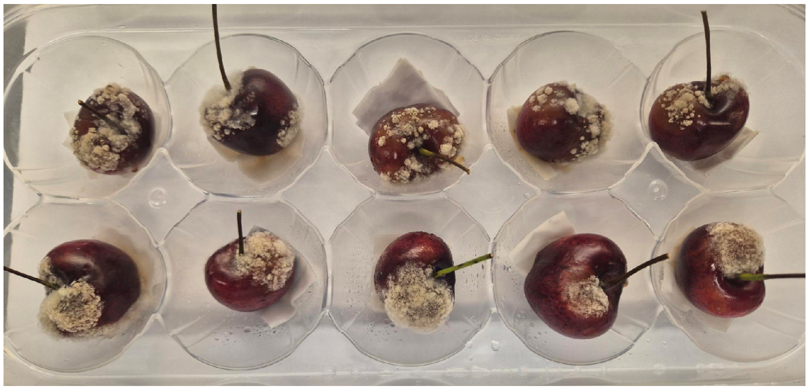

5a

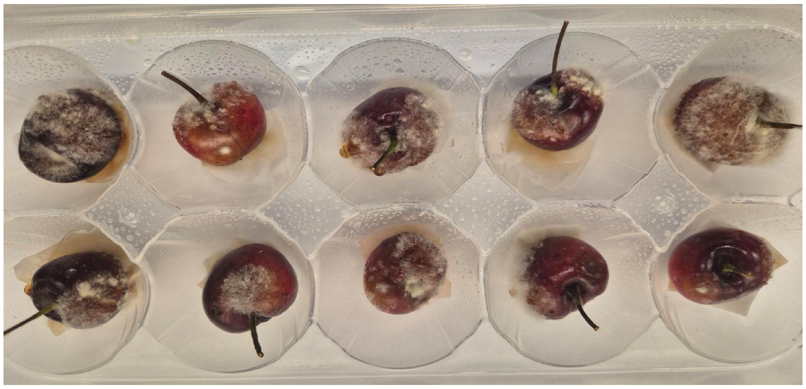

5f

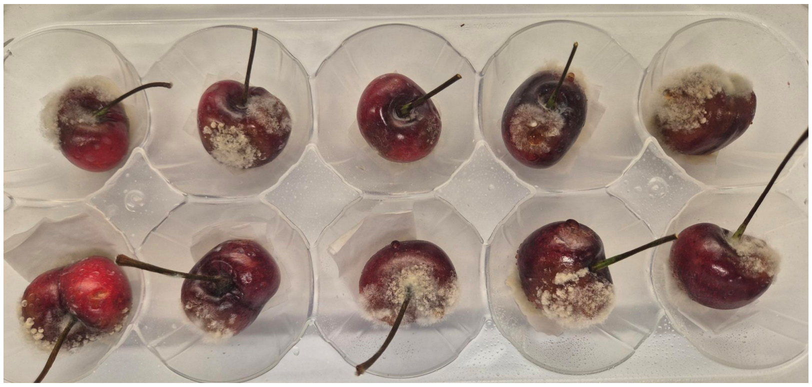

5i

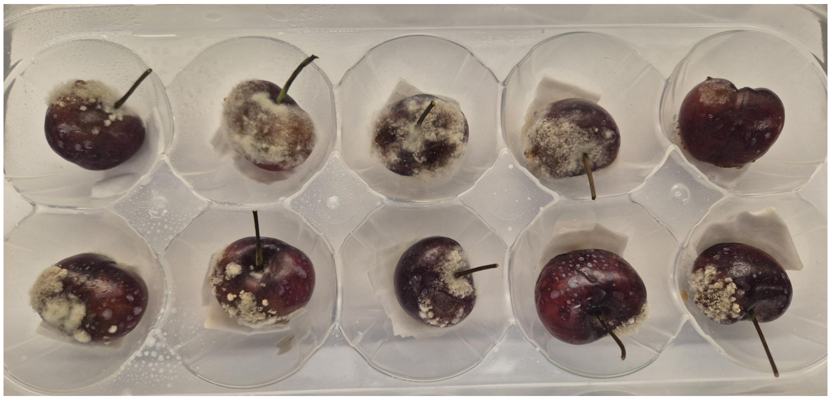

5k

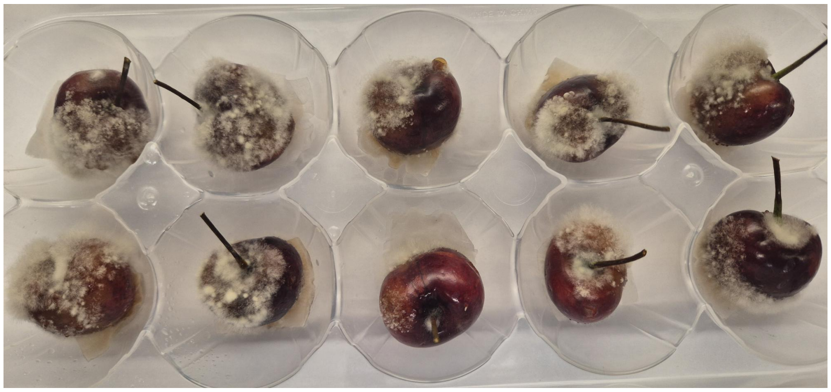

in vivo assay of compound series 6

6

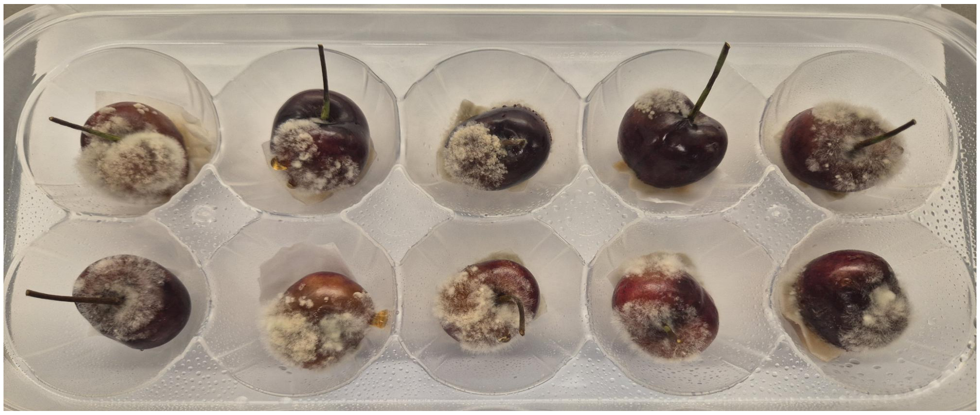

6a

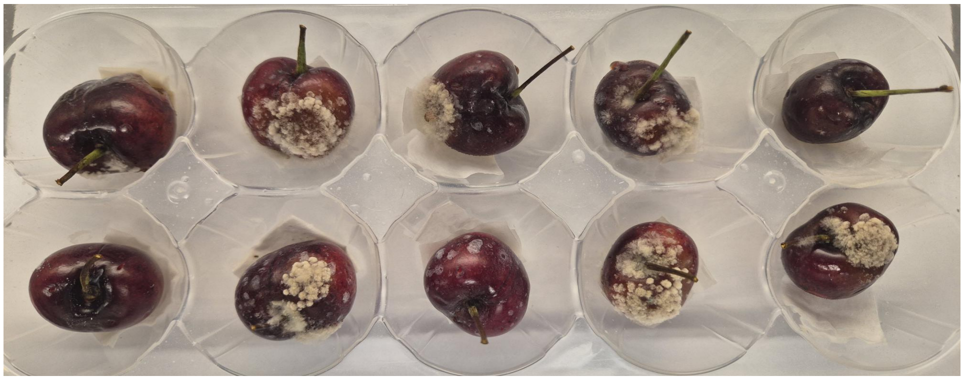

6d

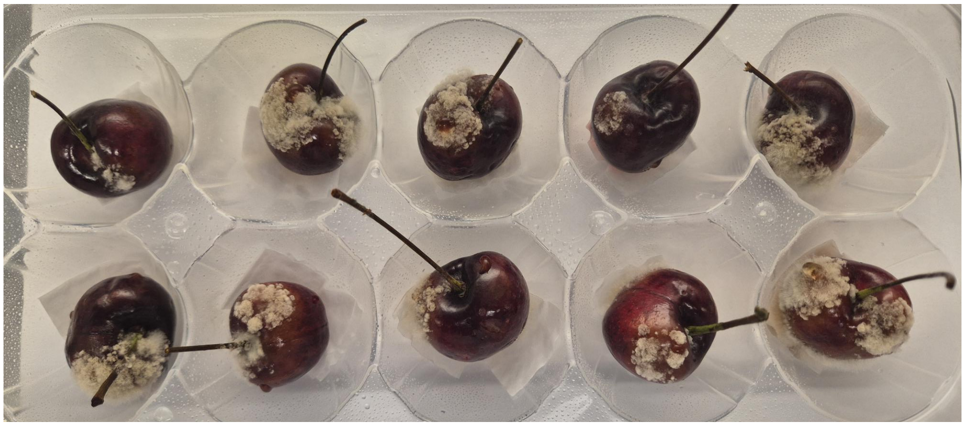

6f

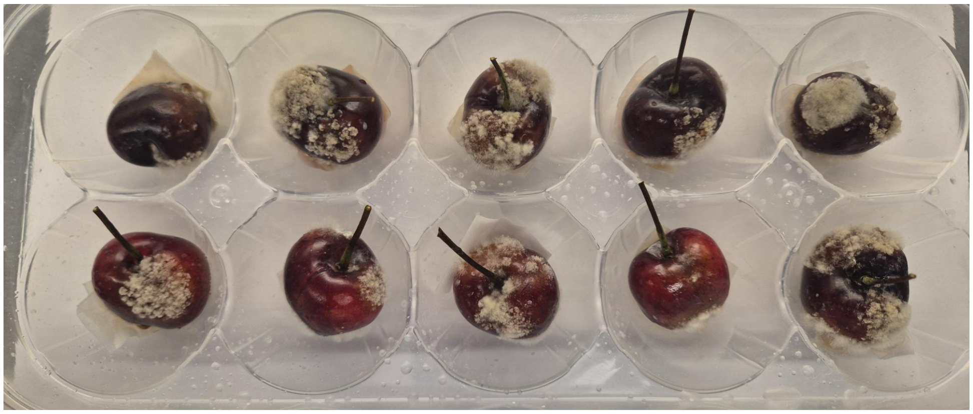

6k

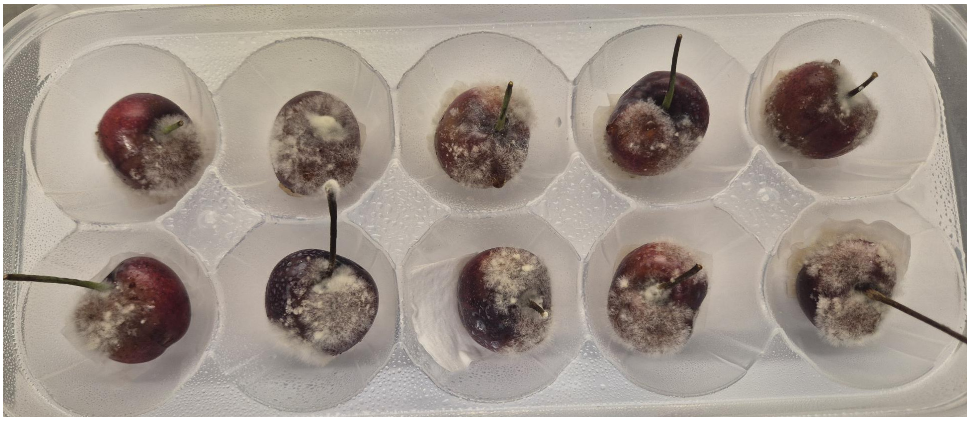

Negative control, water only, without inoculum

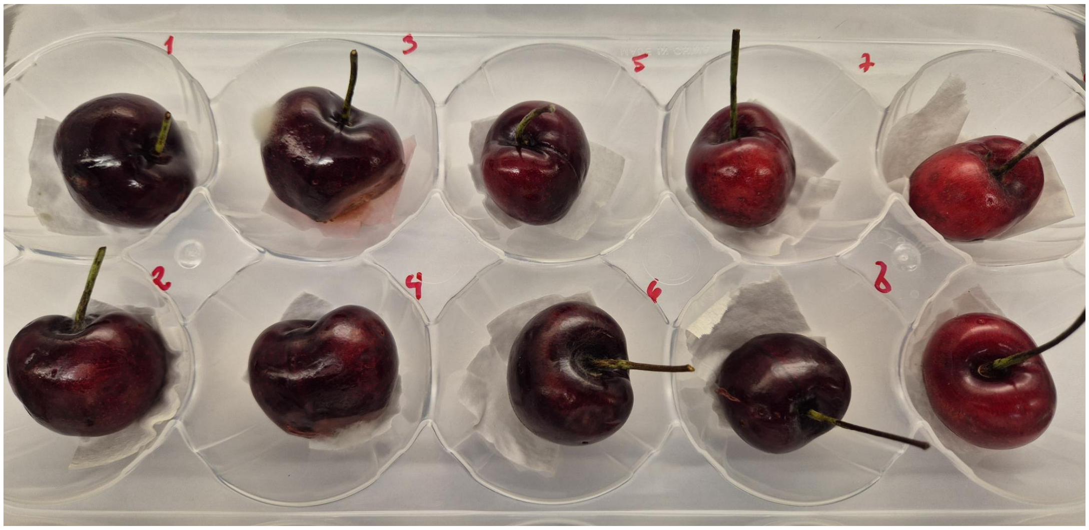

Negative control with inoculum

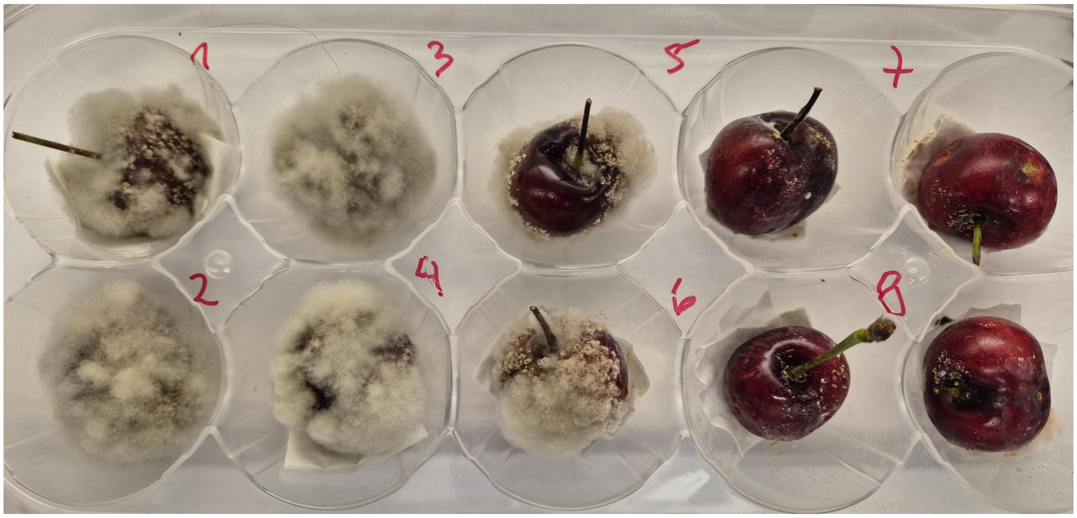

Mystic (0.4 mL/L)

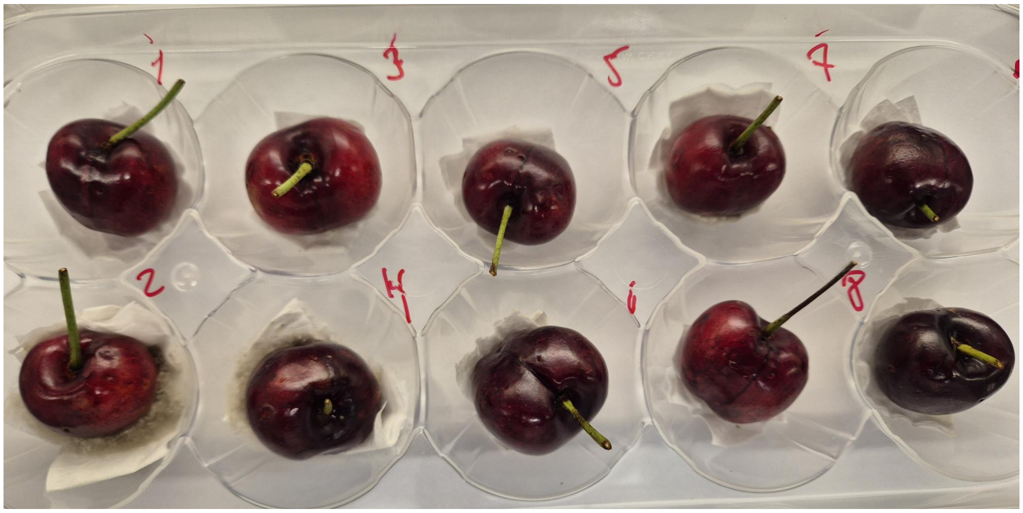

Captan (1.8 g/L)

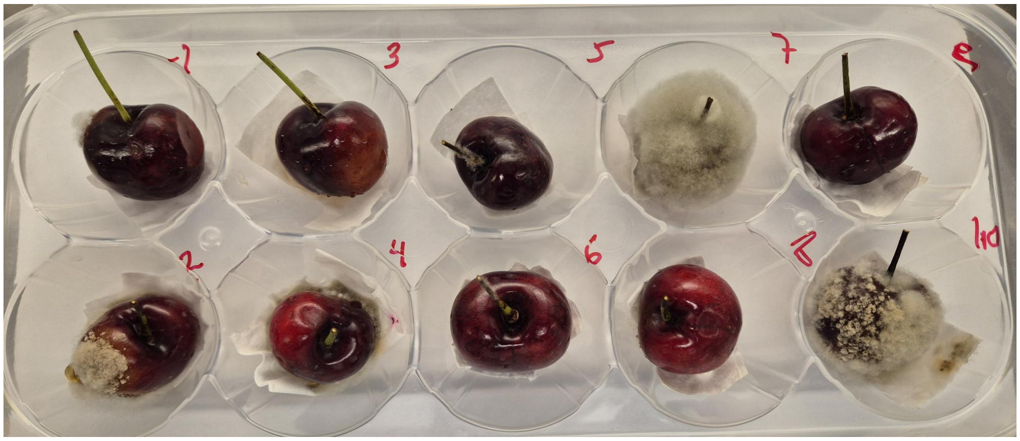
1
3
5
7
2
4
6
8
10

Table S1. Physicochemical descriptor values used for principal component analysis, along with corresponding PC1 and PC2 scores for *B. cinerea*.

ID	pEC50	MolWt	cLogP	TPSA	HBD	HBA	RotBonds	PC1	PC2
4c	3.895	266.14	3.91	32.86	1	1	3	0.658	0.034
4e	3.773	280.17	4.30	32.86	1	1	4	1.389	0.652
4f	3.772	280.17	4.16	32.86	1	1	3	0.968	0.339
4k	3.695	334.60	4.81	32.86	1	1	2	1.640	1.050
5	4.913	196.05	2.93	15.79	1	0	0	-3.436	0.179
5i	3.680	330.18	4.17	42.09	1	2	3	2.782	-0.951
6	4.935	196.05	2.93	15.79	1	0	0	-3.436	0.179
6a	4.007	238.08	3.13	32.86	1	1	1	-0.803	-1.202
6d	3.747	266.14	3.77	32.86	1	1	2	0.237	-0.279

Table S2. Physicochemical descriptor values used for principal component analysis, along with PC1 and PC2 scores for *M. fructicola*.

ID	pEC_{50}	MolWt (g/mol)	cLogP	TPSA	HBD	HBA	RotBonds	PC1	PC2
4	5.152	196.05	2.93	15.79	1	0	0	-3.673	0.449
4c	4.602	266.14	3.91	32.86	1	1	3	1.019	-0.636
4d	4.511	266.14	3.77	32.86	1	1	2	0.533	-0.575
4f	4.776	280.17	4.16	32.86	1	1	3	1.306	-0.282
4g	3.700	300.16	4.16	32.86	1	1	2	1.100	0.105
4k	4.913	334.60	4.81	32.86	1	1	2	1.834	1.018
5	4.889	196.05	2.93	15.79	1	0	0	-3.673	0.449
5a	4.271	238.08	3.13	32.86	1	1	1	-0.527	-1.223
5f	4.433	280.16	4.16	32.86	1	1	3	1.306	-0.282
5k	3.619	334.60	4.81	32.86	1	1	2	1.834	1.018
6	4.725	196.05	2.93	15.79	1	0	0	-3.673	0.449
6a	4.498	238.08	3.13	32.86	1	1	1	-0.527	-1.223
6f	3.943	280.17	4.16	32.86	1	1	3	1.306	-0.282
6k	3.737	334.60	4.81	32.86	1	1	2	1.834	1.018

Table S3. Experimental reaction parameters (temperature and time) for 4-bromoindole derivatives (**4a–k**).

Compound	Temperature (°C)	Time (min)
**4a**	70	10
**4b**	80	10
**4c**	80	5
**4d**	80	5
**4e**	80	15
**4f**	90	15
**4g**	120	20
**4h**	100	20
**4i**	120	20
**4j**	90	40
**4k**	90	30
